# Supplementary material for: Charting γ-secretase substrates by explainable AI
Source: Nat Commun. 2025 Jul 1;16:5428. doi: 10.1038/s41467-025-60638-z (PMC12219630; doi:10.1038/s41467-025-60638-z)
Supplement: Supplementary file 1 — Supplementary Information [file 41467_2025_60638_MOESM1_ESM.pdf]

Supplementary Information

Charting γ-secretase substrates by explainable AI

Stephan Breimann[1,2,3,*], Frits Kamp[1,*], Gabriele Basset[1], Claudia Abou-Ajram[1], Gökhan Güner[2,4], Kanta Yanagida[5,6], Masayasu Okochi[6], Stephan A. Müller[2,4], Stefan F. Lichtenthaler[2,4,7], Dieter Langosch[8], Dmitrij Frishman[3,#] & Harald Steiner[1,2,#]

[*], These authors contributed equally to this work
[#], To whom correspondence should be addressed: dmitrij.frischman@tum.de (D.F.), harald.steiner@med.uni-muenchen.de (H.S.)

[1] Biomedical Center (BMC), Division of Metabolic Biochemistry, Faculty of Medicine, LMU Munich, München, Germany
[2] German Center for Neurodegenerative Diseases (DZNE), DZNE Munich, München, Germany
[3] Department of Bioinformatics, Technical University of Munich (TUM), Freising, Germany
[4] Neuroproteomics, School of Medicine and Health, TUM University Hospital, München, Germany
[5] Department of Pharmacotherapeutics II, Faculty of Pharmacy, Osaka Medical and Pharmaceutical University, Takatsuki, Japan
[6] Neuropsychiatry, Department of Integrated Medicine, Division of Internal Medicine, Osaka University Graduate School of Medicine, Suita, Japan
[7] Munich Cluster for Systems Neurology (SyNergy), München, Germany
[8] Biopolymer Chemistry, TUM, Freising, Germany

Tables of Contents

Supplementary Figures 2
List of Supplementary Figures 2
Supplementary Methods 21
Data preparation 21
Feature engineering via Comparative Physicochemical Profiling (CPP) 23
Substrate prediction by machine learning 28
Explainable AI 34
Functional bioinformatics analysis of γ-secretase substrates 37
Supplementary Algorithms 40
Supplementary References 41

Supplementary Figures

List of Supplementary Figures

Supplementary Fig. 1 | CPP feature and algorithm ... 3
Supplementary Fig. 2 | Two examples of CPP features ... 4
Supplementary Fig. 3 | dPULearn algorithm and identified non-substrates ... 5
Supplementary Fig. 4 | Benchmarking of CPP and dPULearn ... 6
Supplementary Fig. 5 | Substrate prediction using machine learning ... 7
Supplementary Fig. 6 | Comparison of dataset-annotation combinations for substrate prediction ... 9
Supplementary Fig. 7 | Cell-based experimental validation of predicted substrate and non-substrate candidates ... 10
Supplementary Fig. 8 | Cell-free γ-secretase cleavage analysis of 'minimal substrates' ... 11
Supplementary Fig. 9 | Explainable AI analysis by combining CPP with SHAP ... 12
Supplementary Fig. 10 | Fuzzy labeling demonstrated by TREM2 ... 14
Supplementary Fig. 11 | CPP-SHAP analysis for SLC27A1 ... 15
Supplementary Fig. 12 | CPP-SHAP analysis for four proteins from distinct confidence-based substrate classes ... 16
Supplementary Fig. 13 | Network modules for new HC substrates ... 17
Supplementary Fig. 14 | Integration of network modules and pathway clusters for new HC substrates ... 18
Supplementary Fig. 15 | Functional bioinformatics analysis of γ-secretase substrate associations ... 19

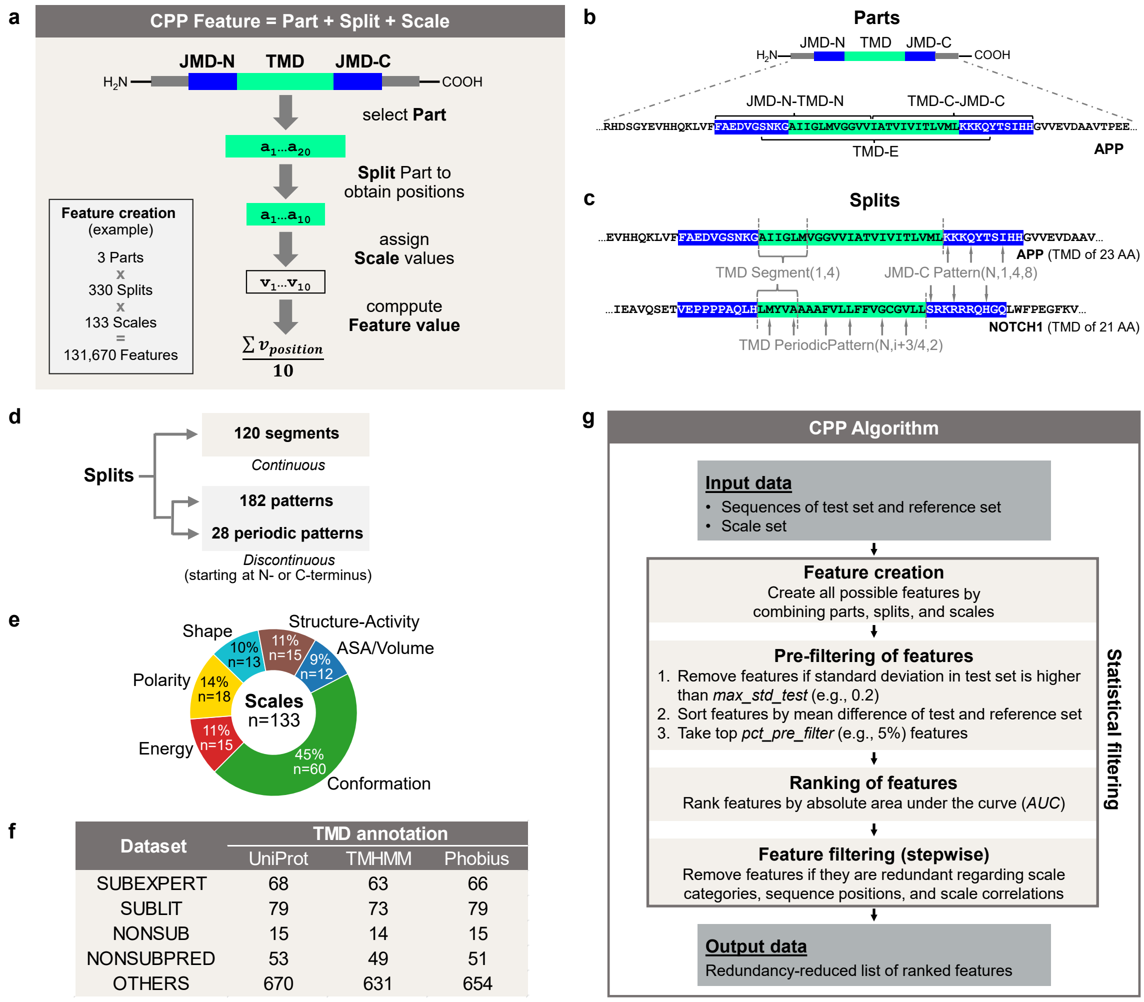

Supplementary Fig. 1 | CPP feature and algorithm. a Concept of the Comparative Physicochemical Profiling (CPP) feature, defined as a part-split-scale combination. The selected part (e.g., TMD) is split to obtain specific sets of amino acids. Values of a given physicochemical scale are assigned to these amino acids and the mean is computed (feature value). **b** Sequence parts for single-span transmembrane proteins, exemplified for TMHMM-based annotation of APP. **c** The three split types (segment, pattern, and periodic pattern) illustrated by two sequences with different TMD length: TMHMM-based annotation of APP and UniProt-based annotation of NOTCH1 (murine sequence). **d** Default splits of CPP. **e** Non-redundant set of physicochemical scales, obtained by AAclust[31] and classified as in AAontology[29]. The best scale set (see Supplementary Methods 'Derivation of the optimal scale and part sets for CPP') contained 133 scales from 6 different scale categories, such as Conformation (green) or Energy (red). **f** Overview of datasets used for feature engineering and machine learning for the three different TMD annotations of UniProt, TMHMM, and Phobius. Two substrate datasets, expert-curated (SUBEXPERT) and literature-based (SUBLIT); two non-substrates datasets, literature-based (NONSUB) and a prediction-based (NONSUBPRED) obtained by dPULearn; and a reference dataset containing proteins with unknown substrate status (OTHERS). **g** Flowchart of the CPP algorithm, comprising feature creation, pre-filtering of features, feature ranking, and feature filtering. Using a test (e.g., SUBEXPERT) and a reference (e.g., OTHERS) set of proteins with a scale set as input, CPP generates over 100,000 features by creating all possible part-split-scale combinations. These features are pre-filtered by different statistical measures (e.g., *mean_dif*) and ranked by the absolute value of the adjusted area under the curve (*AUC*) measure. A stepwise filtering procedure then selects a non-redundant set of features that are most discriminative between the test and reference set (see Supplementary Methods 'CPP algorithm'). Source data are provided as a Source Data file.

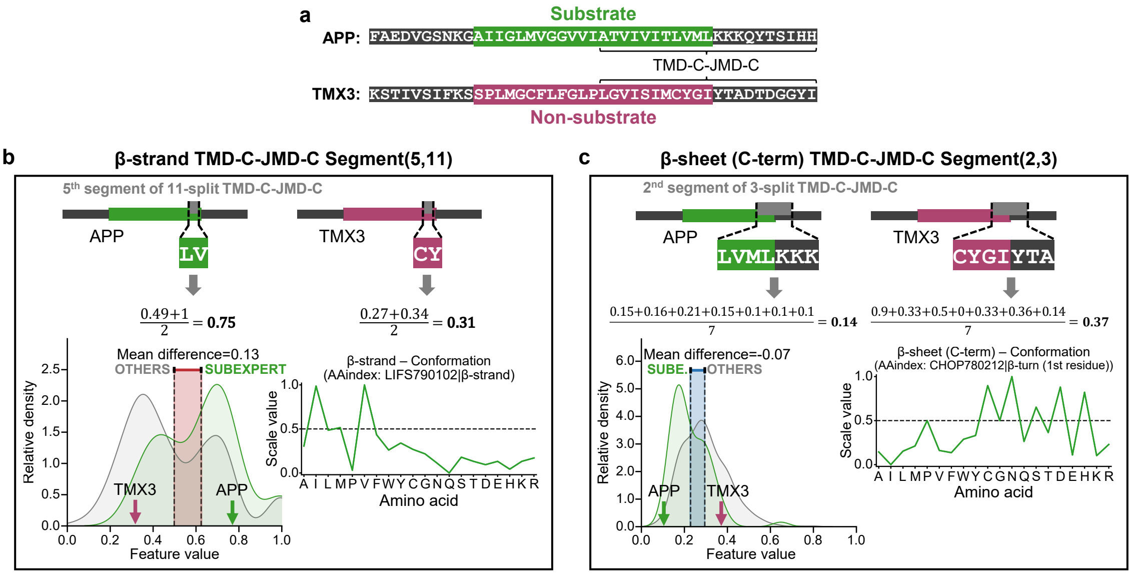

Supplementary Fig. 2 | Two examples of CPP features. a Examples of an established substrate (APP) and a non-substrate (TMX3). TMDs, as annotated by TMHMM, are highlighted by the dataset color code in green (SUBEXPERT) and purple (NONSUB), respectively. **b**, **c** Two features, illustrated by APP and TMX3: 'β-strand TMD-C-JMD-C Segment(5,11)' (**b**) and 'β-sheet (C-term) TMD-C-JMD-C Segment(2,3)' (**c**). Split sequence parts (grey) determine the residue positions to which scale values are assigned for the computation of feature values (bold). The histograms (left) show the distributions of feature values for SUBEXPERT (green) and OTHERS (grey), from which mean differences (*mean_dif*) are derived. Higher feature values for SUBEXPERT yield a positive *mean_dif*, highlighted in red (**b**); lower values result in a negative *mean_dif*, highlighted in blue (**c**). Line plots (right) show the min-max normalized scale values per amino acid. By convention (see Supplementary Methods 'Combining parts, splits, and scales'), scales are given by their subcategory followed here by their category (as defined in AAontology[29]), such as 'β-strand – Conformation' (**b**) and 'β-sheet (C-term)' (**c**). The AAindex scale id and scale name are given in parentheses.

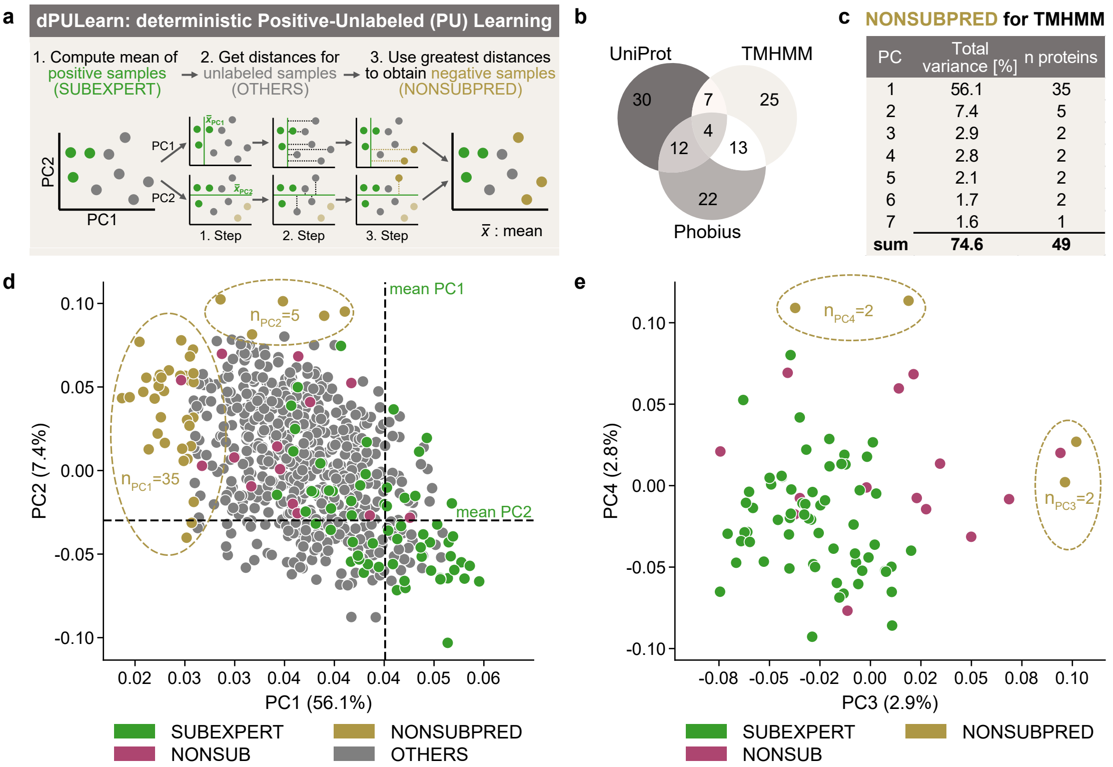

Supplementary Fig. 3 | dPULearn algorithm and identified non-substrates. a Overview of the deterministic Positive-Unlabeled (PU) Learning (dPULearn) algorithm, aiming at identifying additional non-substrates for different principal components (PCs) of a compressed feature space. For each PC, the mean of all positive samples (proteins in SUBEXPERT, green) is computed (step 1), the distance for all unlabeled samples (proteins in OTHERS, grey) is determined (step 2), and samples with the greatest distance are selected as additional non-substrates (gold brown) (step 3). The set of all additional non-substrates is referred to as NONSUBPRED. **b** Venn diagram comparing the sets of identified additional non-substrates for three different TMD annotations (TMHMM, UniProt, and Phobius). **c** Total variance and the number of proteins in NONSUBPRED for the first 7 PCs given for TMHMM annotation. **d**, **e** Scatterplots showing PU learning results for PCs 1–2 (**d**) and 3–4 (**e**) based on TMHMM annotation. Sequence datasets maintain the same color coding as in (**a**). The number of proteins identified based on the *i*-th component is indicated by n_{PCi} (gold brown), corresponding to (**c**). For the first two PCs (**d**), proteins of OTHERS and the means of the positive samples (dashed lines) are given. Source data are provided as a Source Data file.

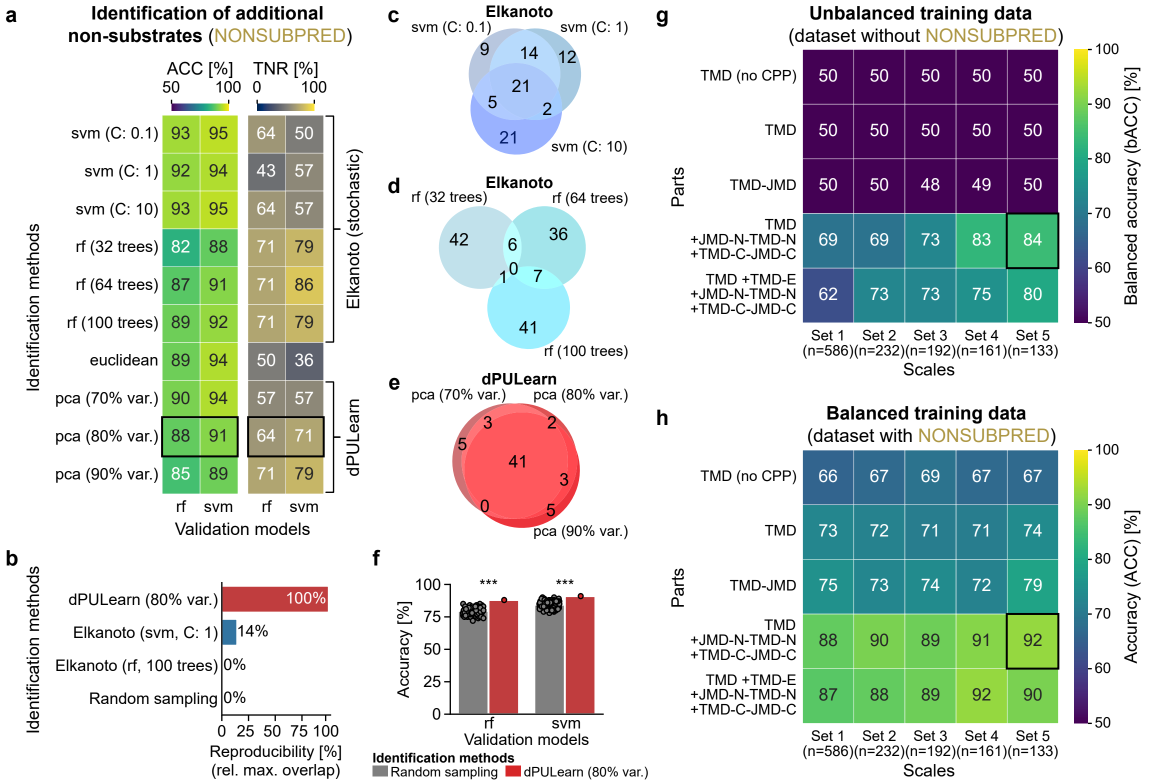

Supplementary Fig. 4 | Benchmarking of CPP and dPULearn. a Heatmap comparing dPULearn against the established PU learning approach by Elkan & Noto (denoted as Elkanoto) using stochastic random forest (rf) and support vector machine (svm) models (see Supplementary Methods 'Benchmarking dPULearn'). Different model parameters were tested, such as the number of trees for random forest. Identified sets of additional non-substrates were used to train two different validation models (random forest and support vector machine) and validated by accuracy (ACC) and true negative rate (TNR). Results for the selected dPULearn approach (pca with 80% variance) are highlighted in bold squares. **b** Bar chart showing the reproducibility of dPULearn compared against Elkanoto and random sampling. **c**–**e** Venn diagrams for sets of identified non-substrates obtained by Elkanoto models (**c**, **d**) and dPULearn (**e**). **f** Bar chart comparing the performance of validation models trained on sets of additional non-substrates identified by dPULearn and random sampling, tested by one-sample t-test (***P<0.001). **g**, **h** Heatmaps showing benchmarking results for CPP and dPULearn based on TMHMM annotation. As baseline models, support vector machines with default settings were used, varying in the features provided by CPP. Five different sets of parts and scales were combined as CPP input. In **g**, models were trained on the imbalanced training dataset (SUBEXPERT vs NONSUB) and evaluated using balanced accuracy (bACC). In **h**, models were trained on the balanced dataset (SUBEXPERT vs NONSUB plus NONSUBPRED obtained by dPULearn) and evaluated using accuracy (ACC). The part and scale set combination with the best performance is highlighted in bold squares. Source data are provided as a Source Data file.

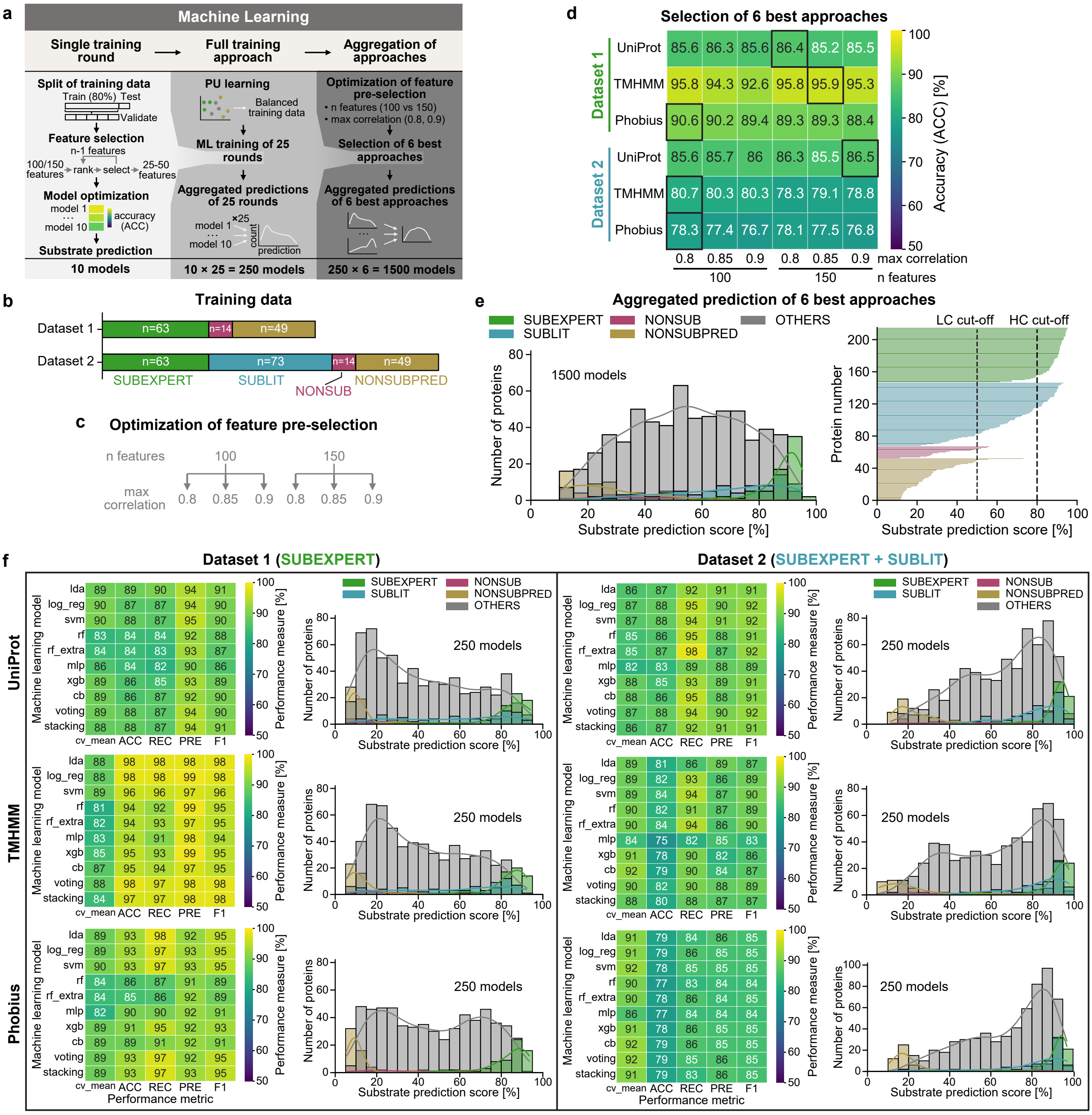

Supplementary Fig. 5 | See next page for caption.

Supplementary Fig. 5 | Substrate prediction using machine learning. a Machine learning workflow. Each training round begins with random splitting of the training dataset, followed by stepwise feature selection and hyperparameter optimization for 10 different machine learning (ML) models to compute prediction scores. In a full training approach, the training dataset is first balanced using dPULearn (PU learning), followed by 25 training rounds (250=25×10 trained models) with subsequent aggregation of prediction scores. Finally, the 6 best approaches for different dataset-annotation combinations are selected (1500=6×250 trained models) and their prediction scores are aggregated, referred to as 'substrate prediction score'. **b** Two machine learning training datasets: dataset 1 (without SUBLIT) and dataset 2 (with SUBLIT). **c** Two optimization parameters for pre-selection of features used in the machine learning pipeline: number of CPP features (100 or 150) and maximum Pearson correlation threshold (0.8, 0.85, and 0.9). **d** Heatmap showing the accuracy (ACC) of various training approaches (based on dataset 1 and dataset 2 with three different TMD annotations) optimized for the pre-selection parameters described in (**c**). For each of the 6 dataset-annotation combinations, the approach with the highest ACC is highlighted with a bold square. **e** Aggregated prediction results of the 6 best approaches, comprising 1500 models. The substrate prediction scores for all initial datasets are shown as a histogram (left, including OTHERS) and a bar chart (right, without OTHERS). Dashed lines indicate the low-confidence (LC) and high-confidence (HC) cut-off according to our confidence-based substrate classification (see Supplementary Methods 'Confidence-based substrate classes'). **f** Comparison of prediction results for 6 best dataset-annotation combinations. Heatmaps (left) present evaluation measures—cross-validation mean accuracy (cv_mean), accuracy (ACC), recall (REC), precision (PRE), and F1 score (F1) (see Supplementary Methods 'Evaluation measures')—aggregated over 25 training rounds for the used 10 machine learning model types (Supplementary Data 10). Histograms (right) display the distributions of substrate prediction scores, shown as in (**e**). Source data are provided as a Source Data file.

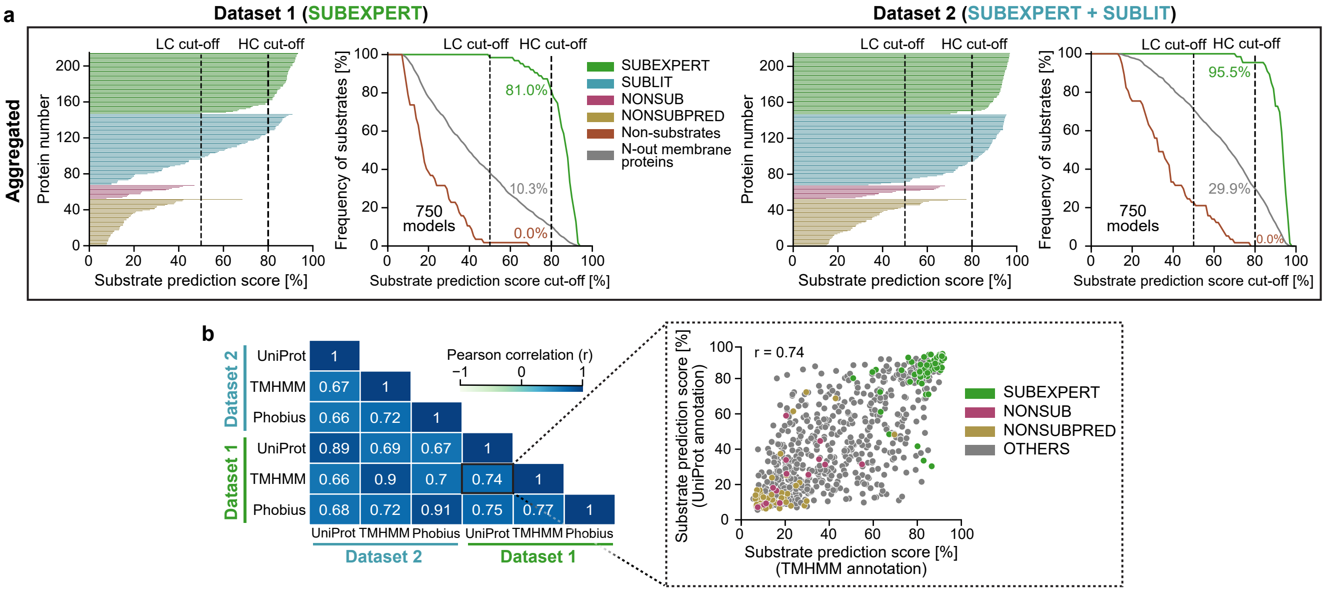

Supplementary Fig. 6 | Comparison of dataset-annotation combinations for substrate prediction. a Aggregation of training approaches over three different TMD annotations for dataset 1 (left) and dataset 2 (right). Bar charts display the distribution of substrate prediction scores as in Supplementary Fig. 5e. Line plots show the substrate proportion in percentage for increasing substrate prediction score cut-offs, with low-confidence (LC) and high-confidence (HC) cut-offs highlighted as in Supplementary Fig. 5e. **b** Heatmap (left) showing the Pearson correlation of prediction scores compared across training datasets and TMD annotations. The scatterplot (right) provides an example comparing the UniProt vs TMHMM annotation for dataset 1 (Pearson's r=0.74). Predictions for OTHERS were included in the correlation calculations. Source data are provided as a Source Data file.

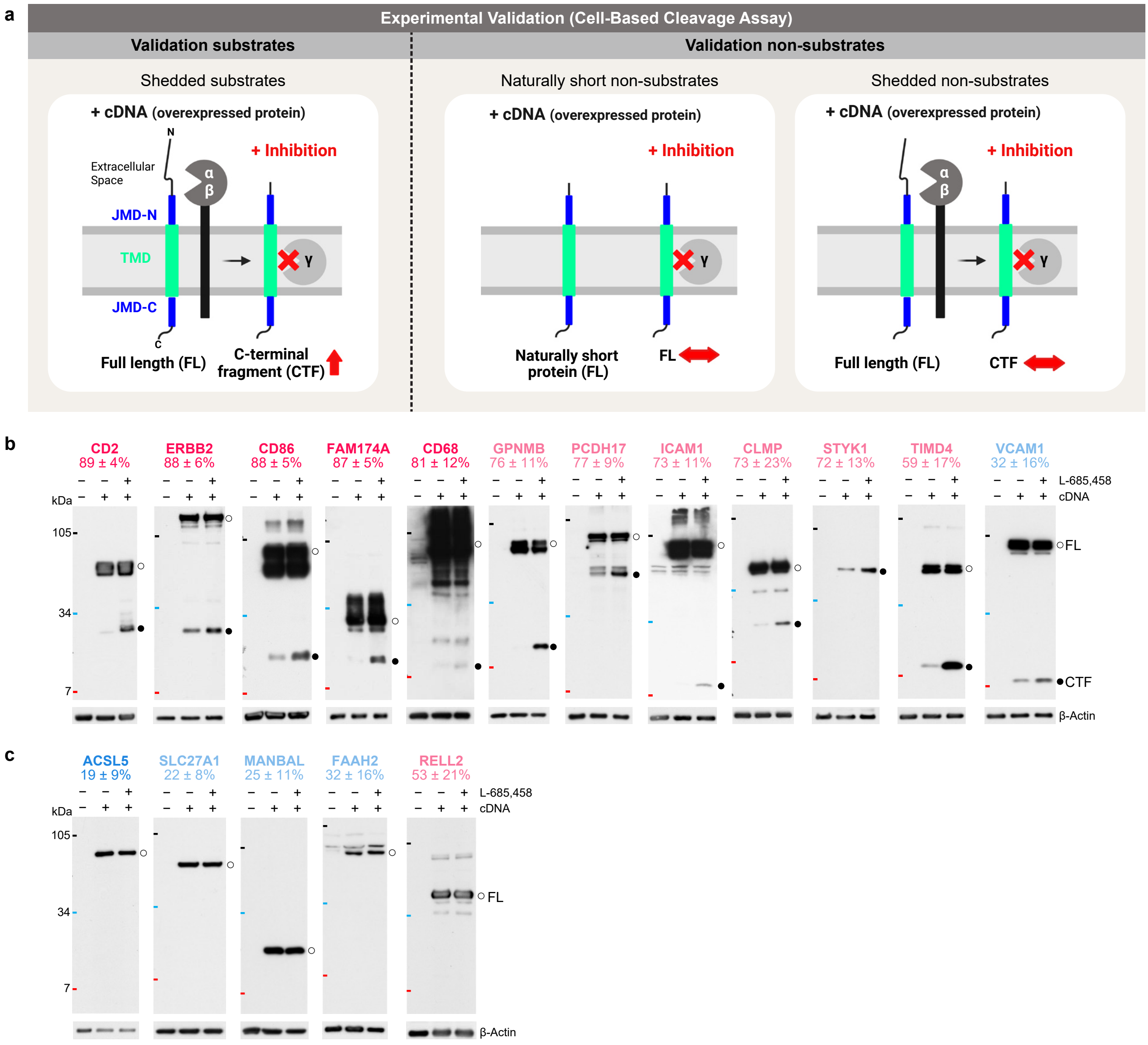

Supplementary Fig. 7 | Cell-based experimental validation of predicted substrate and non-substrate candidates. a Scheme depicting the experimental assessments of the substrate status for different types of candidate proteins expressed in HEK293 cells. Inhibition refers to the use of PS1/2 DKO cells or γ-secretase inhibitor L-685,458 (Created in BioRender. Breimann, S. (2025) https://BioRender.com/ccd3qqc). **b** Immunoblot analysis of substrate candidates. For most proteins, ectodomain-shedding of the full-length (FL) form (○) generates a C-terminal fragment (CTF, ●), which could potentially be cleaved by γ-secretase. Accumulation of the CTFs in the presence of L-685,458 validates their substrate status, as shown in (**a**). For STYK1, the FL protein accumulated as this protein has a naturally short ectodomain, so that STYK1 can be cleaved directly by γ-secretase. For ERBB2, a smaller band than the expected molecular weight of its CTF was found, likely due to subsequent caspase cleavage of the accumulating CTF[64]. β-Actin served as loading control. **c** Immunoblot analysis of non-substrate candidates, performed as in (**b**). All tested non-substrate candidates had a naturally short ectodomain. Levels of their FL form were the same in the presence of L-685,458, showing that these proteins were not cleaved by γ-secretase. Substrates and non-substrates are indicated by their gene name followed by their substrate prediction scores ± standard deviation (see Supplementary Methods 'Aggregation of prediction results') using the same color code for confidence-based substrate classes as in Fig. 4b. All tested candidates were from human, except CD68, ICAM1, STYK1, ACLS5, and SLC27A1, which were from mouse. Immunoblot analyses in **b**, **c** are representative of three independent experiments. Source data are provided as a Source Data file.

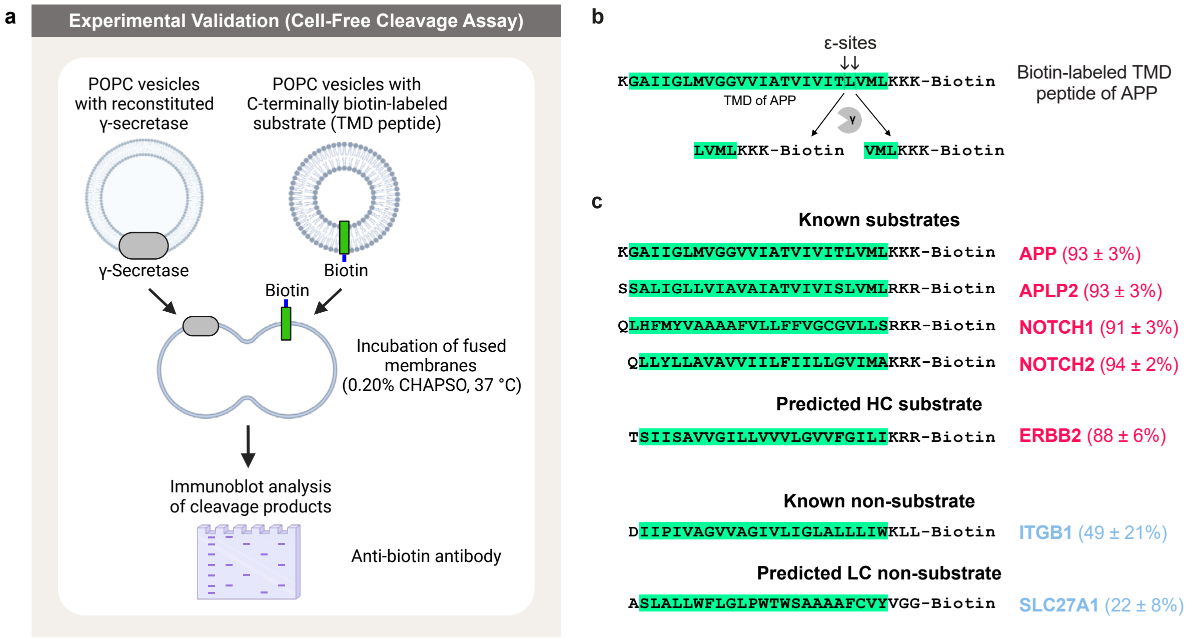

Supplementary Fig. 8 | Cell-free γ-secretase cleavage analysis of 'minimal substrates'. a Workflow of the cell-free cleavage assay. Peptides derived from the transmembrane domain (TMD, green) of substrates and non-substrates are reconstituted in large unilamellar vesicles (LUV) of palmitoyl-oleoyl-phosphatidylcholine (POPC) and then mixed with POPC vesicles containing γ-secretase purified from HEK293 cells. Following overnight incubation, cleavage product formation is analyzed by immunoblotting (Created in BioRender. Breimann, S. (2025) https://BioRender.com/4z2414i). **b** Sequence of the APP-based minimal substrate with expected cleavage products. The ε-sites (↓) indicate the known initial γ-secretase cleavage sites. **c** Sequences of analyzed TMD peptides, with the substrate prediction scores ± standard deviation of the respective protein. TMD annotations (green) were manually curated. Color code for confidence-based substrate classes is as in Fig. 4b. See Fig. 5c, d for results of immunoblot analysis.

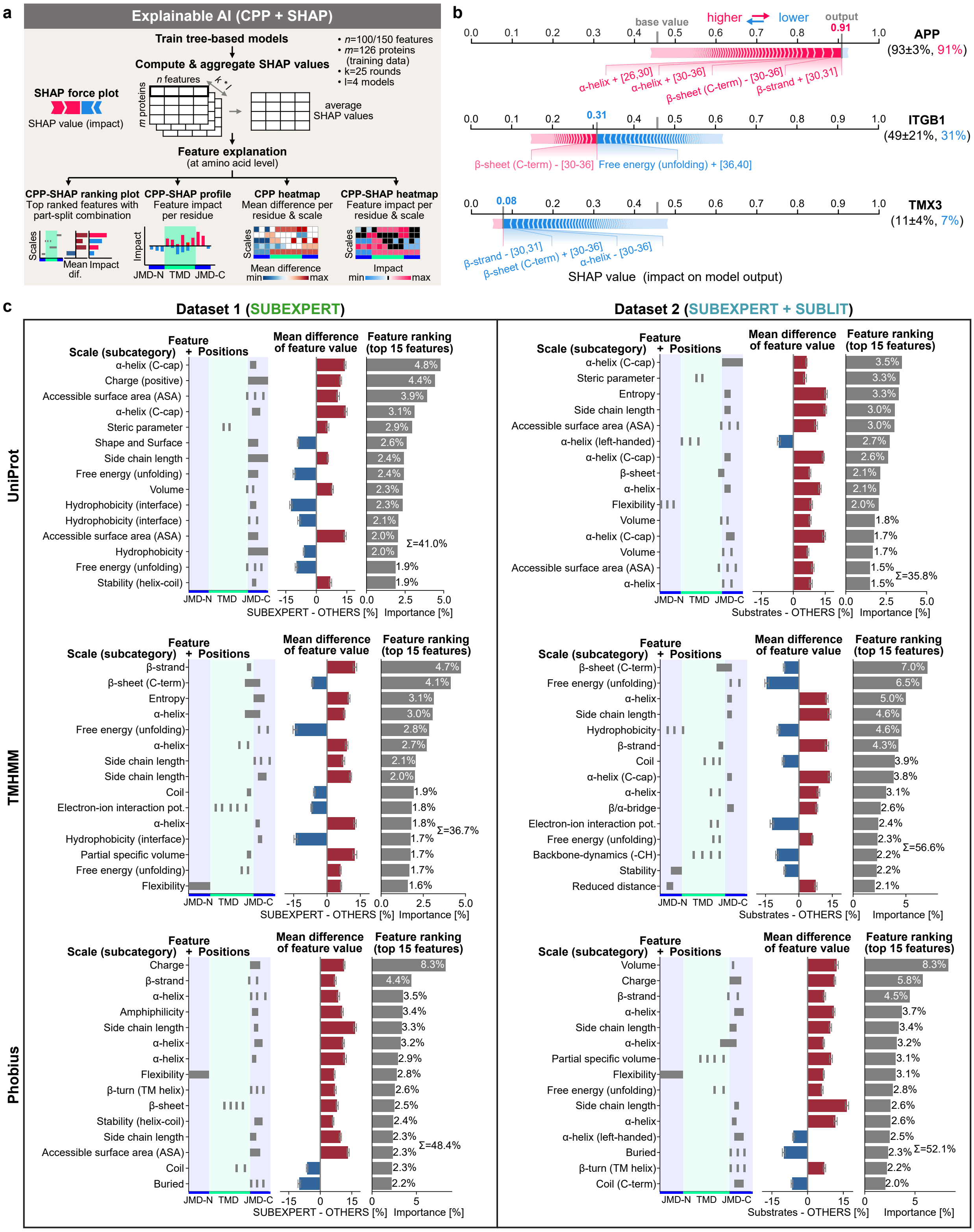

Supplementary Fig. 9 | See next page for caption.

Supplementary Fig. 9 | Explainable AI analysis by combining CPP with SHAP. a Workflow of CPP-SHAP analysis, an explainable AI approach combining Comparative Physicochemical Profiling (CPP) with Shapley Additive exPlanations (SHAP). Four tree-based models are trained on a complete dataset (either dataset 1 or dataset 2) over 25 rounds. SHAP values are computed and aggregated across all rounds and models, yielding average SHAP values for each feature and protein (see Supplementary Methods 'Combining CPP with SHAP'). The prediction results for individual proteins are explained at the amino acid level using four visualizations: 'CPP-SHAP ranking plot', 'CPP-SHAP profile', 'CPP heatmap', and 'CPP-SHAP heatmap' (see Supplementary Methods 'CPP-SHAP plots'). Examples of these four CPP-SHAP plots are shown for ERBB2 (Fig. 7) and SLC27A1 (Supplementary Fig. 11). **b** SHAP force plots illustrating the correspondence between the prediction score (based on dataset 1 and TMHMM annotation) and the SHAP output (i.e., sum of SHAP values). The SHAP output is increased by positive-impact features (red) and decreased by negative-impact features (blue). Three examples of force plots are shown for different confidence-based substrate classes (see Supplementary Methods 'Confidence-based substrates classes'): APP (HC substrate), ITGB1 (LC non-substrate), and TMX3 (HC non-substrate). In the feature name, increased or decreased feature values are indicated by plus and minus signs, respectively. The substrate prediction score ± standard deviation is provided (see Supplementary Methods 'Aggregation of prediction results'), followed by the color-highlighted prediction score (based on TMHMM annotation and dataset 1) that is explained by SHAP. See also CPP ranking plots and CPP-SHAP profiles for APP and ITGB1 in Fig. 6a, d and Fig. 6c, f, respectively. **c** CPP ranking plots showing top 15 features across datasets (dataset 1 and dataset 2) and TMD annotations (UniProt, TMHMM, Phobius). Source data are provided as a Source Data file.

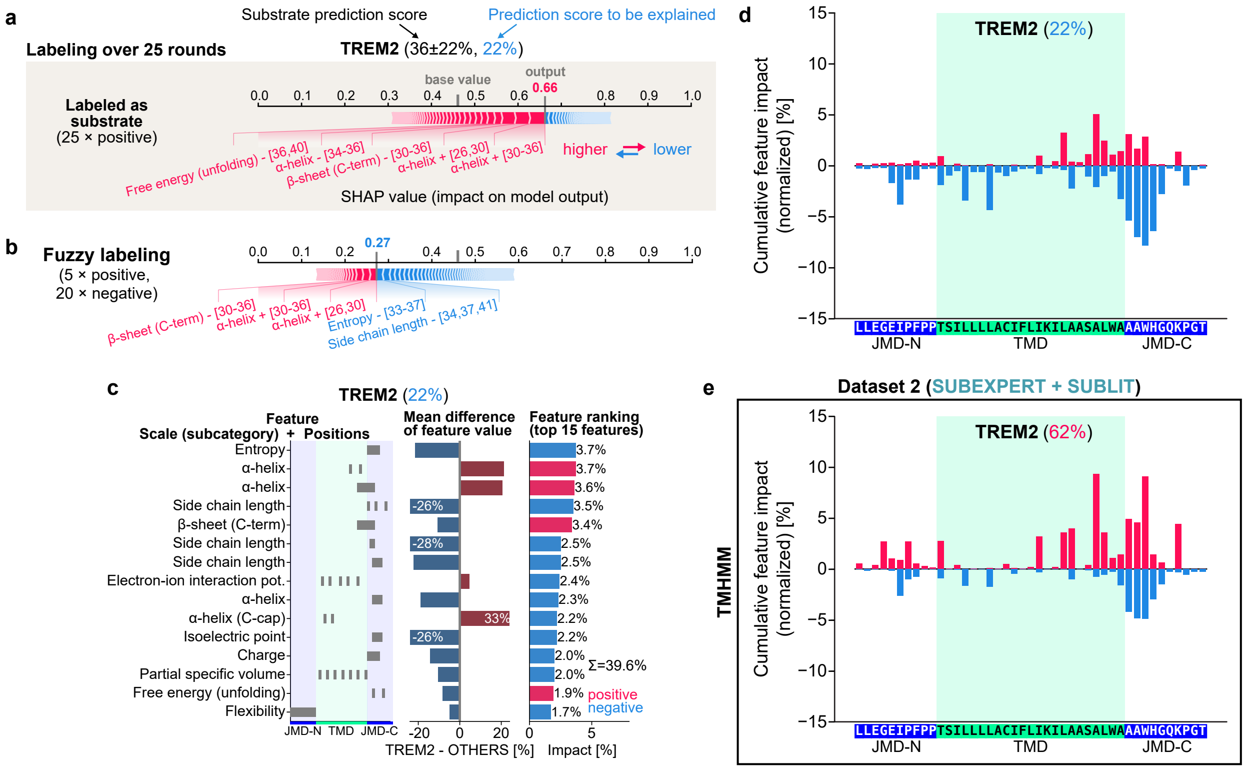

Supplementary Fig. 10 | Fuzzy labeling demonstrated by TREM2. Fuzzy labeling for TREM2 from SUBLIT (36±22% substrate prediction score) to explain its prediction score of 22% (blue) based on the TMHMM annotation and dataset 1. **a**–**d** Two labeling strategies for TREM2 over 25 rounds are compared: (**a**) consistently labeling it as a substrate; and (**b**) applying fuzzy labeling, where TREM2 is labeled 5 times as a substrate and 20 times as a non-substrate, corresponding to its prediction score. Besides the SHAP force plot (**b**), the CPP-SHAP ranking plot (**c**) and CPP-SHAP profile (**d**) illustrate the results of fuzzy labeling for TREM2. Mean difference values in (**c**) are indicated on bars if axis limits are exceeded. **e** CPP-SHAP profile explaining the TREM2 prediction score of 62% based on the TMHMM annotation and dataset 2, in which TREM2 is included (as member of SUBLIT) and therefore labeled as a substrate. Source data are provided as a Source Data file.

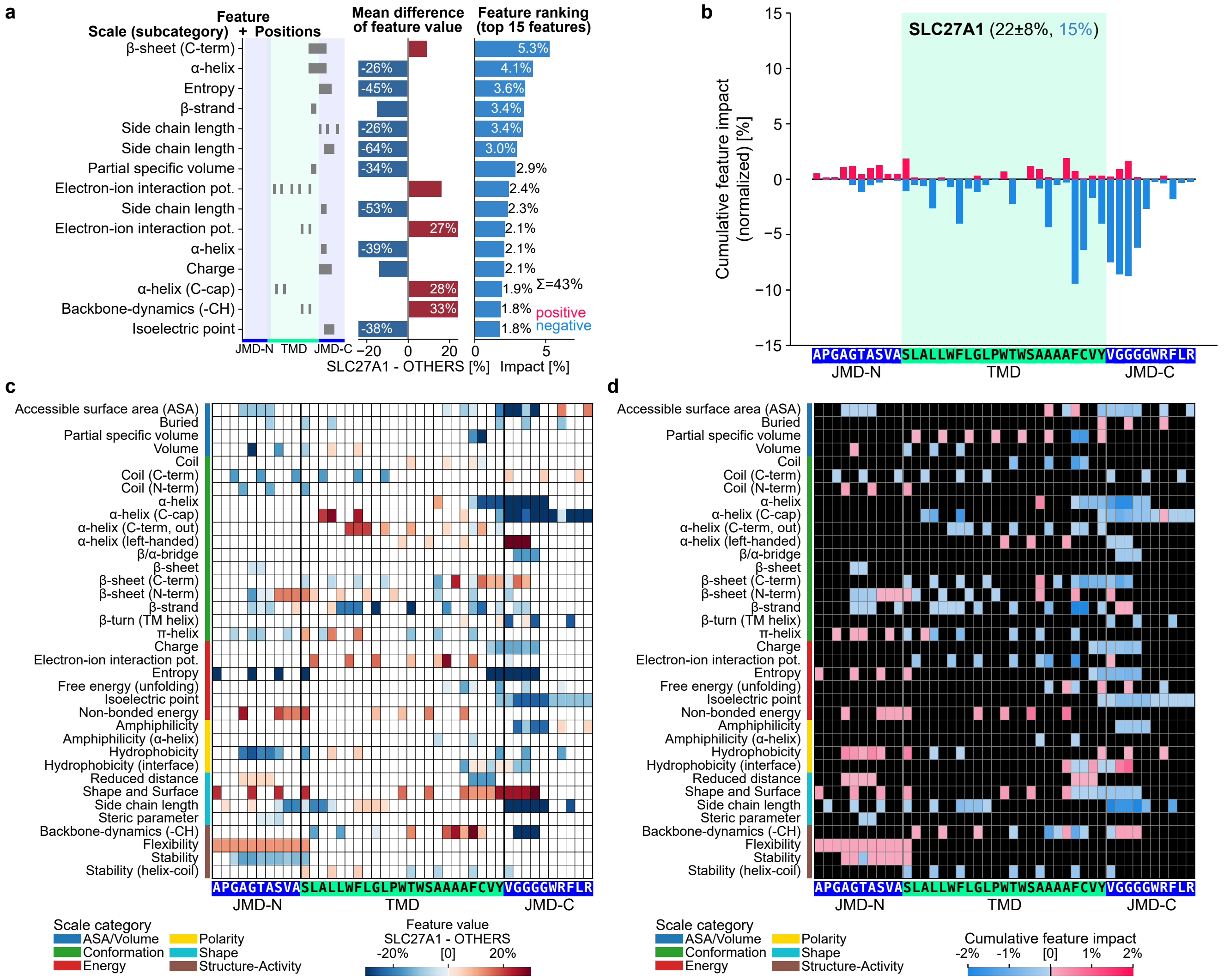

Supplementary Fig. 11 | CPP-SHAP analysis for SLC27A1. The validated LC substrate SLC27A1 analyzed by four CPP-SHAP plots using fuzzy labeling (see Supplementary Methods 'Combining CPP with SHAP'): The CPP-SHAP ranking plot (**a**) ranks the top 15 features by the absolute value of their impact, which can be positive (red) or negative (blue); the CPP-SHAP profile (**b**) shows the cumulative feature impact per residue; the CPP heatmap (**c**) highlights the differences in feature values between the respective protein and the reference dataset (OTHERS) per scale subcategory and residue; and the CPP-SHAP heatmap (**d**) illustrates the feature impact per scale subcategory and residue. Scale categories are from AAontology[29] and uniformly color-coded. The CPP-SHAP analysis results for SLC27A1 (murine sequence, 22±8% substrate prediction score) explain its prediction score of 15% (blue) based on dataset 1 with TMHMM annotation. The CPP-SHAP ranking plot shows exclusively negative-impact features, particularly in the TMD-C and TMD-C anchor, which is further emphasized in the CPP-SHAP profile. The CPP heatmap and CPP-SHAP heatmap disclose the notable negative impact of phenylalanine and cysteine in the TMD-C, attributed to their reduced β-strand tendencies, and of multiple glycines in the TMD-C anchor, resulting from their short side chain length and limited helix termination capacity. For comparison, see the CPP-SHAP analysis for the HC substrate ERBB2 (Fig. 7). Source data are provided as a Source Data file.

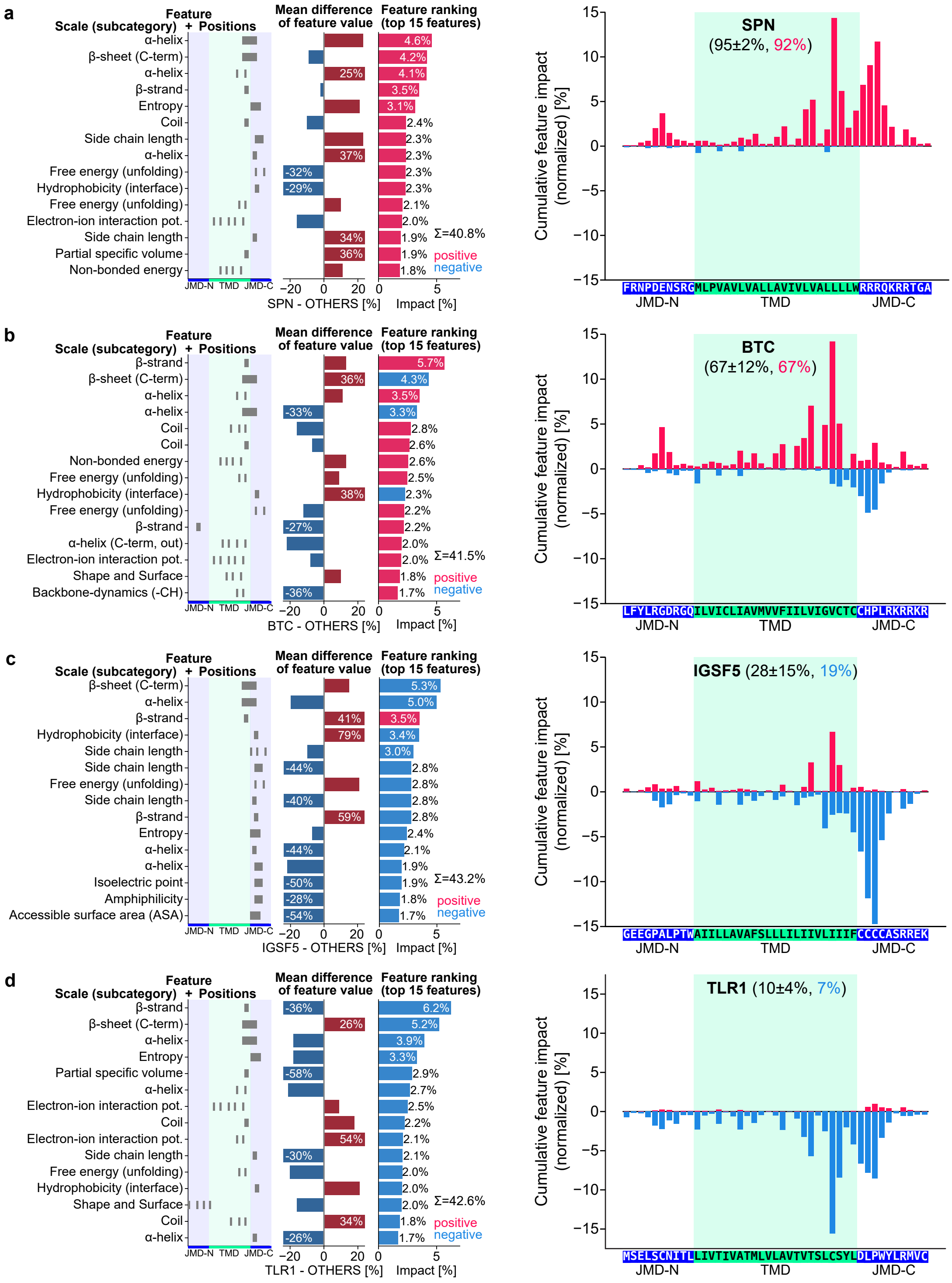

Supplementary Fig. 12 | CPP-SHAP analysis for four proteins from distinct confidence-based substrate classes. CPP-SHAP ranking plots (left) and CPP-SHAP profiles (right) for four selected proteins from Fig. 8b (SPN (**a**), BTC (**b**), IGSF5 (**c**), and TLR1 (**d**)), displayed along the prediction confidence spectrum. Colors indicate positive (red) and negative (blue) feature impact. The substrate prediction score ± standard deviation is provided (see Supplementary Methods 'Aggregation of prediction results'), followed by the color-highlighted prediction score (based on dataset 1 and TMHMM annotation) explained by SHAP. The four selected proteins illustrate the transition from a predominance of positive-impact features in HC substrates, such as SPN (**a**), through a shift toward negative-impact features in LC substrates and LC non-substrates (e.g., BTC (**b**) and IGSF5 (**c**), respectively), to a predominance of negative-impact features in HC non-substrates, such as TLR1 (**d**). Source data are provided as a Source Data file.

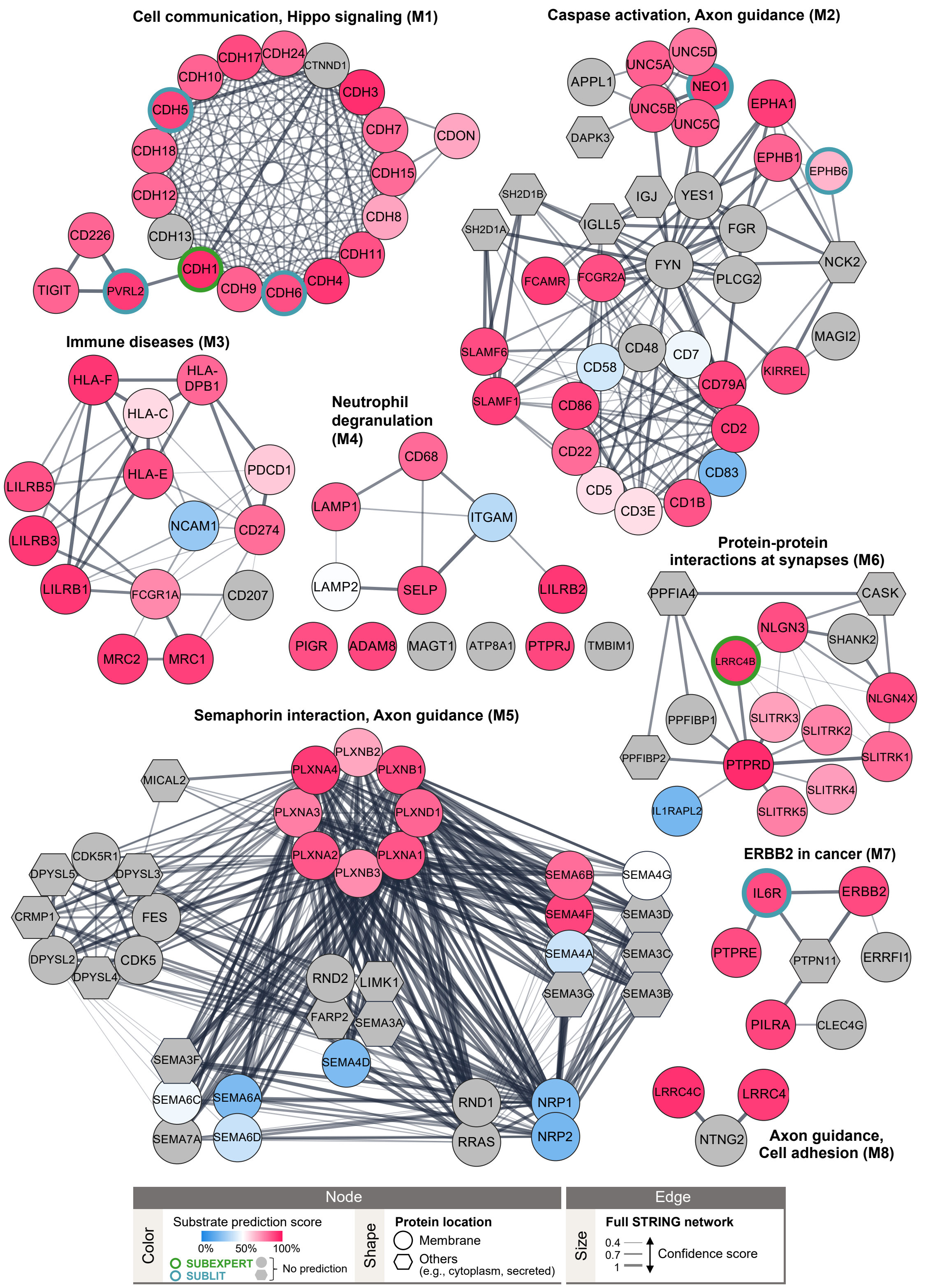

Supplementary Fig. 13 | Network modules for new HC substrates. Map displaying 8 significantly over-represented network modules (M1–M8) of new HC substrates identified by a functional STRING network analysis using DOMINO (see Supplementary Methods 'Network analysis'). Nodes represent proteins, with only those from the human N-out proteome being color-coded according to their substrate prediction score. Edges represent the STRING confidence score of functional gene associations. See Fig. 9a for an overview of the functional bioinformatics analysis. Source data are provided as a Source Data file.

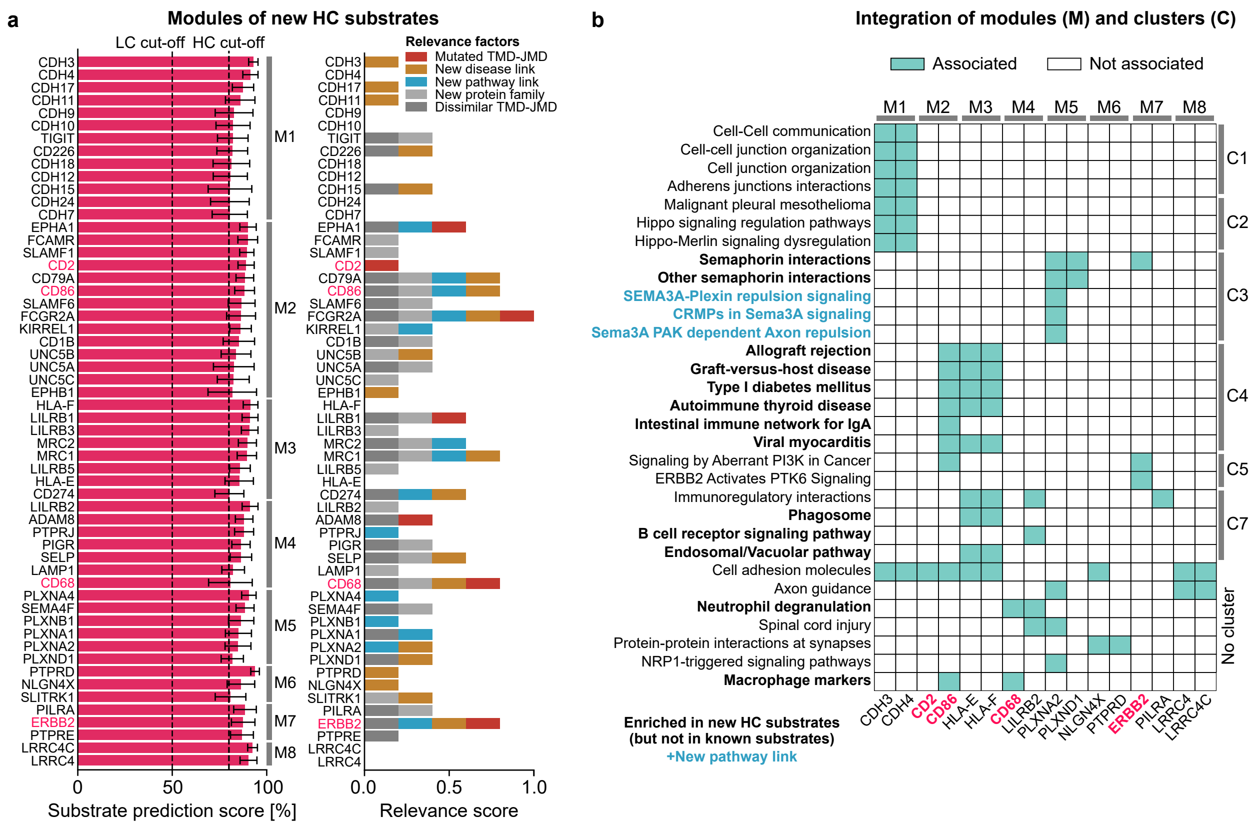

Supplementary Fig. 14 | Integration of network modules and pathway clusters for new HC substrates. a Two bar charts for all new high-confidence (HC) substrates of network modules M1–M8 (Supplementary Fig. 12), showing their substrate prediction score (mean ± standard deviation, left) and relevance score (right), see Supplementary Methods 'Aggregation of prediction results' and 'Computation of relevance score', respectively. Dashed lines indicate low-confidence (LC) and HC cut-offs, with validated substrates highlighted in red. **b** Heatmap displaying the associations (cyan) between pathway terms (Fig. 9e) and selected genes, with grey bars indicating clusters of pathway terms C1–C7 (Fig. 9f) and gene modules M1–M8 (from (**a**)). See Fig. 9a for an overview of the functional bioinformatics analysis. Source data are provided as a Source Data file.

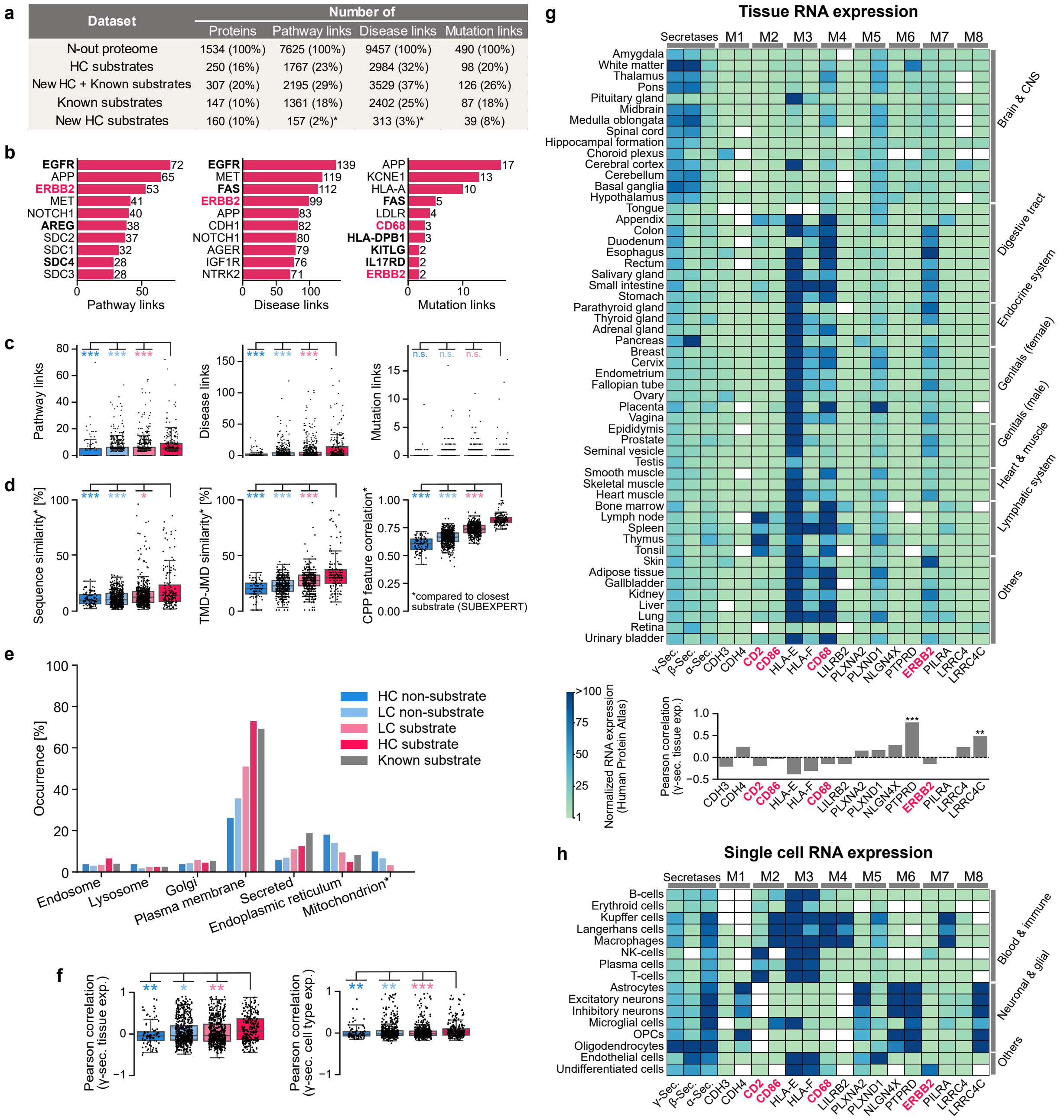

Supplementary Fig. 15 | See next page for caption.

Supplementary Fig. 15 | Functional bioinformatics analysis of γ-secretase substrate associations. a Overview of pathway, disease, and mutation links for different subsets of the human N-out proteome. Counts of pathway and disease links that were not previously associated with γ-secretase or its known substrates are indicated by asterisks. **b** Bar chart ranking the 10 high-confidence (HC) substrates by their pathway, disease, and mutation link counts. New HC substrates are highlighted in bold black, with validated ones in red. **c**, **d** Box plots for association and similarity metrics comparing the four confidence-based substrate classes (see Supplementary Methods 'Confidence-based substrate classes' for sample sizes). Differences between HC substrates and the other classes were tested using two-sided Mann-Whitney-U test with separate Bonferroni correction. Significance levels are indicated by color-coded asterisks (*$P<0.05$, **$P<0.01$, ***$P<0.001$). In **d**, similarity is assessed to the closest (i.e., most similar or most highly correlated) substrate from SUBEXPERT. **e** Subcellular location for four confidence-based substrate classes of human N-out proteome and known substrates. Multiple occurrences are possible. The mitochondrion (asterisk) is an unlikely location of γ-secretase substrates. **f** Box plots for the co-expression analysis of normalized RNA expression values (from Human Protein Atlas) at tissue (left) and single-cell (right) level. Co-expression was assessed by Pearson correlation between γ-secretase and each protein from the four different substrate classes, tested as in (**c**, **d**). **g**, **h** Normalized RNA expression (from (**f**)) for selected proteins (from Supplementary Fig. 14b) at the tissue (**g**) and single-cell (**h**) level. The significance of Pearson correlations between γ-secretase and these proteins was Bonferroni corrected (*$P<0.05$, **$P<0.01$, ***$P<0.001$). Expression level of 0 is indicated in white. Modules M1–M8 are marked by grey bars (top). Tissue and cell groups are adopted from Human Protein Atlas and indicated by grey bars (right). The expression for α-, β-, and γ-secretase is given by the highest expression among ADAM10/17, BACE1/2, and PS1/2, respectively (see Supplementary Methods 'Co-expression analysis'). See Fig. 9a for an overview of the functional bioinformatics analysis. Source data are provided as a Source Data file.

Supplementary Methods

Data preparation

Sequence parts of transmembrane proteins

In this work, we focused on single-span transmembrane proteins (TPs) with an extracellular N-terminal ectodomain (i.e., an N-out topology). These proteins contain an α-helical transmembrane domain (TMD) and can be distinguished by the presence or absence of an N-terminal cleavable signal sequence, referred to as type I and type III, respectively. Type II and type IV denote TPs with an extracellular C-terminal ectodomain (i.e., an N-in topology) and a TMD close to the N- and C-terminus, respectively. The following sequence parts were considered (Supplementary Fig. 1a, b):

- **TMD**: TMD as annotated in the UniProt database[1] or by the transmembrane prediction algorithms TMHMM[2] and Phobius[3].
- **TMD-N/C**: N- and C-terminal halves of TMD.
- **TMD-E**: TMD expanded by 4 amino acid positions on both sides of the membrane. The value of 4 amino acids was chosen according to the length of potential anchoring regions.
- **JMD-N/C**: N-terminal and C-terminal juxtamembrane domains with 10 amino acids.
- **JMD-N-TMD-N**: A combined sequence of JMD-N and TMD-N.
- **TMD-C-JMD-C**: A combined sequence of TMD-C and JMD-C.
- **TMD-JMD**: A combined sequence of JMD-N, TMD, and JMD-C.

We denote the first four residues of the JMD-C of single-span type I TPs as 'TMD-C anchor' (Fig. 2b). Located at the C-terminal cytosolic side of the TMD (Fig. 1a), this region is typically characterized by positively charged residues (Fig. 1b–d) 'anchoring' the TMD at the membrane-water interface by electrostatic interactions with negatively charged phosphate groups[4].

Datasets

A dataset of 4464 single-span TP sequences (2365 from human and 2099 from mouse) was obtained from the UniProtKB/Swiss-Prot database[1] (Supplementary Data 1, 2), with missing UniProt topology information being supplemented by Phobius predictions. Since γ-secretase substrates have an N-out topology and are mainly of type I[5], we kept all type I TPs for further analysis, a total of 2155 (1146 from human and 1009 from mouse). Among these were 126 known substrates from human and mouse[5-8] and 12 known human non-substrates[9-11]. The set of 2155 proteins was extended by 21 known substrates—7 with other topologies (2 type III TPs and 5 single-span TPs lacking UniProt topology information) and 14 single-span type I TPs from other organisms (12 from rat and 2 from chicken). Additionally, 3 known non-substrates with missing UniProt topology information were included, resulting in 2179 proteins. Due to requirements of subsequent analysis steps, sequences with JMDs shorter than 10 amino acids were removed, yielding a total of 2090 proteins.

To create a redundancy-reduced dataset of proteins with unknown substrate status, the CD-HIT algorithm[12] was applied to the relevant sequence parts (TMD-JMD) with a 40% cut-off, yielding clusters of proteins with at least 40% sequence similarity. Clusters containing known substrates or non-substrates were discarded. From each of the remaining clusters, one representative sequence was selected, with preference given to human sequences and plasma membrane proteins, resulting in a set of 670 proteins with unknown substrate status, referred to as OTHERS.

To obtain high-quality training data for machine learning, the set of substrates was manually divided into an expert-curated (SUBEXPERT) and a literature-based (SUBLIT)

substrate dataset, using rigorous selection criteria (see below). We applied CD-HIT with a 60%-similarity cut-off to the substrate dataset. As a result, the following 4 proteins were not considered for SUBEXPERT: NRXN2 and NRXN3 due to their similarity with NRXN1; as well as PTPRK and NLGN2 due to their similarity with PTPRM and NLGN1, respectively.

We obtained the following datasets given for the UniProt annotation (followed by the n numbers for the TMHMM annotation in parenthesis):

- **SUBSTRATES:** Set of 147 (n=136 for TMHMM) known γ-secretase substrates (91 from human, 42 from mouse, 12 from rat, and 2 from chicken) including 144 substrates from the most recent review[5] and 3 additional substrates (ADAM10[7], SYT7[8], RYK[6]). The review contains 149 substrates, but a few adjustments for substrate selection were made for various reasons (Supplementary Data 1).
- **SUBEXPERT**: Non-redundant subset of SUBSTRATES with 68 (n=63) expert-curated γ-secretase substrates selected if convincing and/or multiple evidence for cleavage were reported in ref. [5] and the corresponding primary literature. Evidence criteria were accumulation of endogenous or overexpressed substrates and/or reduction of substrate cleavage product formation in the presence of γ-secretase inhibitors, active-site mutants or genetic knock-out of the catalytic presenilin subunit (PS1 or PS2) of γ-secretase. In addition, knowledge of cleavage product functions (e.g., signaling of the intracellular domains), identification of the cleavage sites, and/or in vitro studies of enzyme kinetics were considered.
- **SUBLIT**: Subset of SUBSTRATES with 79 (n=73) literature-based γ-secretase substrates that were not selected for SUBEXPERT.
- **NONSUB**: Non-redundant set of 15 (n=14) γ-secretase non-substrates, experimentally identified by proteomic studies[9,10] or a genome-wide screen[11]. The following 6 non-substrates with known sheddase (i.e., protease cleaving N-terminal ectodomain) were selected from refs. [10,11] (BCAM, F11R, LMAN1, SEMA4B, TMX3, PGFRA). 7 proteins with a short ectodomain, not requiring shedding prior to γ-secretase cleavage, were provided by Lichtenthaler (FXYD3, MTDH, PMEPA1, PTTGIP, reported in ref. [13]; RELL1, TGFA, TYROBP, unpublished data). Data from ref. [14] also indicate a non-substrate status of TYROBP. Furthermore, based on ref. [9] we included 2 non-substrates (ITGB1, NPR1), in which no CTF accumulation was detected in the presence of a γ-secretase inhibitor using a HEK293 cell overexpression system.
- **OTHERS**: Non-redundant set of 670 (n=631) single-span type I TPs (551 from human and 119 from mouse) for which it is unknown whether they are substrates or non-substrates of γ-secretase. Proteins have a sequence similarity of less than 40% with sequences from SUBEXPERT, SUBLIT, and NONSUB.
- **NONSUBPRED**: Set of 53 (n=49) predicted non-substrates that are reliably identified from OTHERS using our deterministic positive-unlabeled (PU) learning algorithm (see 'Computational non-substrate identification by dPULearn').

These datasets were generated based on the TMD annotations by UniProt as well as by TMHMM and Phobius predictions. TMHMM and Phobius mostly yielded a somewhat smaller dataset because of either missing TMD predictions or too short JMDs due to alternative TMD annotations. For example, the protein Klotho occurs only in SUBEXPERT based on the TMD annotation by UniProt. A summary of the datasets for the three TMD annotation approaches is given in Supplementary Fig. 1f and Supplementary Data 1. See Supplementary Data 2 for the SUBSTRATES, NONSUB, and OTHERS datasets as well as Supplementary Data 11 for the NONSUBPRED dataset. Unless stated otherwise, the TMHMM annotation was used because

it allowed for the best prediction results (see 'Aggregation of prediction results', Supplementary Fig. 5d).

Since most γ-secretase substrates require shedding (i.e., removal of their N-terminal ectodomain) prior to γ-secretase cleavage, we compiled two lists of substrates for the two main sheddase families: the 'A Disintegrin And Metalloprotease' (ADAM) family, commonly referred to as α-secretases, as well as the 'β-site of APP cleaving enzyme 1' (BACE1) and BACE2 family, termed β-secretases. Substrate lists for α-secretases and β-secretases are provided in Supplementary Data 3, 4, respectively.

One further dataset was collated for a functional bioinformatics analysis:

- **Human N-out proteome**: A set of all 1534 human single-span N-out (type I or type III) TPs with a JMD length of at least 10 amino acids each (Supplementary Data 17).

For training machine learning models, we assembled the following two datasets (given for the TMHMM annotation) of substrates and non-substrates used as positive and negative samples, respectively:

- **dataset 1**: 63 substrates from SUBEXPERT as well as 63 non-substrates from NONSUB and NONSUBPRED.
- **dataset 2**: 136 substrates from SUBEXPERT and SUBLIT as well as 63 non-substrates from NONSUB and NONSUBPRED.

Sequence logos

We used the WebLogo server[15] to create sequence logos for three sequence datasets. The first set comprised 23 γ-secretase substrates with known initial endoproteolytic cleavage site, which is approximately located 4–5 amino acids at the N-terminal side relative to the TMD-C anchor. The substrates were aligned based on their cleavage site. The second and third dataset, both based on the TMHMM annotation, comprised 63 substrates from SUBEXPERT and 14 non-substrates from NONSUB, respectively.

Feature engineering via Comparative Physicochemical Profiling (CPP)

Idea of the CPP algorithm

Comparative Physicochemical Profiling (CPP) is a sequence-based feature engineering algorithm to identify the most distinctive features between two sets of protein sequences. The core idea of CPP is its representation of features, referred to as CPP features. CPP extends and amalgamates sequence segmentation techniques[16] with dis-/continuous motif identification[17] and *n*-gram methods, which split sequences in overlapping or non-overlapping segments of *n* adjacent residues[18]. A CPP feature is defined as a combination of a part, a split, and a scale (Supplementary Fig. 1a). CPP first splits a sequence part, such as the TMD, into smaller segments or patterns. Scale values are then assigned to each residue, and their mean values are computed. These means are used for a statistical comparison between the two protein datasets.

By exhaustive generation of all possible part-split-scale combinations, CPP creates over 100,000 features and filters them statistically down to a user-defined number (default 100) of non-redundant features (Supplementary Fig. 1a; see Fig. 2a for the general workflow including CPP). CPP features are highly interpretable and facilitate explainable feature engineering, which leads to transparent and expressive machine learning models[19-21].

Splitting of sequence parts
Sequence parts, such as TMD or TMD-C-JMD-C (Supplementary Fig. 1b), can be split into segments, patterns, or periodic patterns (Supplementary Fig. 1c). In the following, any set of parts can be considered.

Segments: Each part was split into between 1 and 15 continuous segments. For a particular split, an i-th segment is denoted as Segment(i:n), where n is the number of segments and $i \in [1, 2 \ldots n]$, counting from the N-terminal end. For example, splitting a part of length L=20 into 4 segments (n=4) would result in the following four equally sized segments of length 5: Segment(1,4), Segment(2,4), Segment(3,4), and Segment(4,4). If the part length is not divisible by n, we used a simple rounding procedure to obtain segments of approximately equal length (Supplementary Algorithm 1).

Patterns: Each part was split into discontinuous patterns containing between 2 and 4 amino acids separated by 3 or 4 amino acid positions, which roughly corresponds to the distance between the amino acids located on the same face of an α-helix[22]. A pattern can start either at the N- or C-terminus of a part. In the latter case, amino acid positions are counted backwards, starting from the C-terminus. For patterns starting at the N-terminus and consisting of 2, 3, and 4 amino acids we use the notation Pattern(N,y1,y2), Pattern(N,y1,y2,y3), and Pattern(N,y1,y2,y3,y4), respectively, with y1, y2, y3, and y4 denoting the positions of amino acids with respect to the N-terminus of a given part. Analogously, for patterns starting at the C-terminus, the notation is Pattern(C,y1,y2), Pattern(C,y1,y2,y3), and Pattern(C,y1,y2,y3,y4), respectively, with y1, y2, y3, and y4 denoting the positions of amino acids counting from the C-terminus of a given part. For example, Pattern(N,1,4,8) comprises the 1st, the 4th, and the 8th amino acid of a part counting from the N-terminus.

Periodic patterns: Each part was split into periodic patterns consisting of every 3rd, 4th, or alternating 3rd and 4th amino acids within a whole part. In general, a periodic pattern represents a face of an α-helix[22] or other conformational relations of residues that are close but not directly adjacent to each other. A periodic pattern can start either at the N- or at the C-terminus of a part beginning at an amino acid position *start* between 1 and 4. For periodic patterns starting at the N-terminus, we use the notation PeriodicPattern(N,i+y1/y2,*start*), where y1 denotes the 1st and every further odd amino acid position, while y2 denotes the 2nd and every further even amino acid position. For example, PeriodicPattern(N,i+3/3,1) represents a discontinuous pattern consisting of every 3rd amino acid starting at the 1st N-terminal amino acid. Likewise, PeriodicPattern(N,i+3/4,2) represents a discontinuous pattern starting at the second amino acid with alternating every 3rd and 4th amino acid (see example below). Analogously, for periodic patterns starting at the C-terminus, the notation PeriodicPattern(C,i+y1/y2,*start*) is used, with y1 and y2 denoting amino acid positions as before but counting from the C-terminus of a given part.

By splitting sequence parts into segments, patterns, and periodic patterns, sequences of different length can be compared. For example, APP has a TMD length of 23 residues (TMHMM annotation) whereas the TMD of NOTCH1 has 21 residues (UniProt annotation) (Supplementary Fig. 1c). By convention, 'TMD Segment(1,4)' means for APP the first 6 amino acids of its TMD ('AIIGLM'), while for NOTCH1 just the first 5 residues ('LMYVA'). Likewise, 'JMD-C Pattern(N,1,4,8)' gives a discontinuous sub-sequence comprising the 1st, 4th, and 8th amino acid within the JMD-C ('K-Q-I' and 'S-R-H' for APP and NOTCH1, respectively). The periodic pattern 'TMD PeriodicPattern(N,i+3/4,2)' yields for APP a periodic sub-sequence with 7 residues ('I-L-G-I-I-T-L'), while comprising 6 residues for NOTCH1 ('M-A-F-L-G-V').

In total, CPP creates 330 splits, comprising 120 segments, 182 patterns, and 28 periodic patterns (Supplementary Fig. 1d). Examples of all CPP part-split combinations for APP and TMX3 are given in Supplementary Data 5.

Classification of scales

We assembled an initial set of 652 amino acid physicochemical property scales, comprising 566 scales from the AAindex database[23], 72 accessible surface area (ASA) scales from[24], and 14 hydrophobicity scales from ref. [25]. The latter two datasets were included because ASA is a key feature for determining protein folding[26] and side-chain conformation[27], while hydrophobicity is highly relevant for assessing TMD backbone dynamics[22] and helical unfolding[28]. After removing completely redundant scales and those containing missing values, we obtained a dataset of 586 amino acid scales, including 553, 21, and 12 scales from AAindex, Lins et al. [24] and Koehler et al. [25], respectively.

Each scale was min-max normalized to the [0,1] range as $x'_i = (x_i - min(\boldsymbol{x}))/(max(\boldsymbol{x}) - min(\boldsymbol{x}))$, where $\boldsymbol{x}$ is a vector containing numerical values for the 20 canonical amino acids, x_i is the original value for one amino acid, and x'_i is its normalized value. Property scales were classified into 8 categories and 67 subcategories, as obtained from AAontology[29]: ASA/Volume (64 scales, 5 subcategories), Composition (58, 5), Conformation (224, 24), Energy (36, 9), Others (17, 6), Polarity (111, 6), Shape (45, 6), and Structure-Activity (31, 6). 42 scales could not be assigned to a specific subcategory and were therefore included in 'unclassified' subcategories, defined for each corresponding category.

Selection of scales

To derive a redundancy-reduced set of amino acid scales, we created five scale sets. Set 1 contained all 586 scales mentioned above, while the remaining 4 sets were redundancy-reduced subsets of Set 1, assembled in two steps. First, scale sets were selected based on different scale classification criteria. To obtain a redundancy-reduced version of Set 1, Set 2 was kept in this step identical to Set 1. Unclassified scales and scales from scale category 'Others' were removed for Sets 3-5 and, additionally, all scales from the scale category 'Composition' were removed from Sets 4 and 5. To minimize redundancy due to anti-correlated scales (e.g., hydrophilicity *vs* hydrophobicity), scales assigned to selected subcategories were removed, where the selection for Set 4 was less strict than for Set 5 (Supplementary Data 6). Thus, before redundancy reduction, the scale sets comprised the following numbers of scales and subcategories: Set 1 (586 scales, 67 subcategories), Set 2 (586, 67), Set 3 (532, 62), Set 4 (415, 52), Set 5 (353, 42).

Subsequently, redundancy-reduced subsets of Sets 2–5 were obtained by agglomerative clustering[30] (employing the complete linkage method) in conjunction with our AAclust framework[31]. Other linkage methods (average, single, Ward) and *k*-means clustering were tested and performed worse. AAclust selects one representative scale for each cluster and can work with a user-defined number of clusters *k*. We optimized *k* such that each selected scale subcategory was contained at least once in the scale set (Supplementary Algorithm 2). As a result, we received 586, 232, 192, 161, and 133 scales for Sets 1-5, respectively (Supplementary Data 7). In summary, Set 1 is a redundant set of 586 propensity scales, whereas Set 2–5 are redundancy-reduced subsets of Set 1. Set 5 was chosen for subsequent analysis (Supplementary Fig. 1e) because it allowed for the best performance (Supplementary Fig. 4g). See Supplementary Data 12 for evaluation results of CPP with various AAclust scales selections.

Combining parts, splits, and scales

CPP features are part-split-scale combinations and follow a strict naming convention. Splitting parts results in a distinct set of amino acids, with each amino acid being described in terms of property scales. For example, 'β-strand TMD-C-JMD-C Segment(5,11)' describes a feature in

which a specific property scale—tendency of amino acids to form a β-strand conformation—is considered for the amino acids of the 5th segment of the TMD-C-JMD-C sequence part that is split into 11 segments, starting from the N-terminus. Next, the average scale value over all residues of this segment is computed, referred to as feature value. Consider the sequences of APP and the known non-substrate TMX3 (Supplementary Fig. 2a). The part-split combination of 'TMD-C-JMD-C Segment(5,11)' results in short sub-sequences for APP ('LV', its initial cleavage site) and TMX3 ('CY'). By choosing a β-strand scale (AAindex: LIFS790102), the CPP feature of 'β-strand TMD-C-JMD-C Segment(5,11)' results in the feature value of 0.75 for APP and 0.31 for TMX3 (Supplementary Fig. 2b). This means that APP has a high β-strand propensity around its initial cleavage site, while this propensity is low for TMX3 at the corresponding position. Scales are given by their subcategory, obtained from AAontology, to enable a more uniform data integration in subsequent analysis steps. For instance, consider the feature 'β-sheet (C-term) TMD-C-JMD-C Segment(2,3)' (Supplementary Fig. 2c), where the AAindex scale CHOP780212 describes the propensity of a residue to occur at the 1st position of a β-turn. This scale was assigned to the 'β-sheet (C-term)' subcategory due to its correlation with other scales assigned to the same subcategory.

CPP algorithm

We seek to delineate those features that exhibit the most significant differences between the test dataset of well-established γ-secretase substrates (SUBEXPERT) and a reference dataset (OTHERS). Taking two datasets of protein sequences (test dataset T and reference dataset R) and a set of property scales as input, the CPP algorithm (Supplementary Fig. 1g) involves four steps:

1. **Feature creation**: CPP creates a set of features for all possible combinations of given parts, splits, and scales. For instance, 100,000 features are generated given 4 parts, 250 splits, and 100 scales. For all features, feature values (i.e., average scale value over all amino acids for a part-split combination) are computed for each protein of T and R.
2. **Pre-filtering**: Features are pre-filtered such that only a user-defined percentage *pct_pre_filter* of all created features remains. For each feature, CPP calculates the standard deviation of the feature values in the test dataset *std*(T) and the mean feature values in T and R to determine their difference. This mean difference is defined by:

$$mean\_dif(T,R) = \bar{T} - \bar{R}$$

   where $\bar{T}$ and $\bar{R}$ are given by $\bar{T} = \frac{1}{n_T} * \sum_{i=1}^{n_T} v_i$ and $\bar{R} = \frac{1}{n_R} * \sum_{i=1}^{n_R} v_i$ with n_T and n_R indicating the number of all proteins in T and R, respectively, and v_i denoting the feature value of the i-th protein. Next, features are removed for which $std(T)$ is higher than a certain threshold called *max_std_test*. Given the total number of features n_{total}, CPP selects the best $n = n_{total} * pct\_pre\_filter$ features with the highest absolute values of *mean_dif*(T, R). For instance, CPP obtains 5000 features for n_{total} = 100,000 and *pct_pre_filter*=0.05.
3. **Ranking**: For each remaining feature, CPP uses the area under curve (AUC) as a non-parametric measure of the difference between T and R [32], computed as follows:

$$AUC = \frac{1}{n_T n_R} \sum_{i}^{n_T} \sum_{j}^{n_R} k(x_{i1} x_{j2})$$

   where n_T and n_R are the numbers of proteins in T and R, respectively, x_{i1} and x_{j2} are the feature values for the i-th protein in T and the j-th protein in R, respectively, and the

indicator function k for comparing two feature values is defined as:

$$k(x_{i1}, x_{j2}) = \begin{cases} 0 & \text{if } x_{i1} < x_{j2} \\ 0.5 & \text{if } x_{i1} = x_{j2} \\ 1 & \text{if } x_{i1} > x_{j2} \end{cases}$$

Finally, the *AUC* is adjusted by subtracting 0.5 such that it ranges between -0.5 and 0.5, i.e., between the extreme cases that all feature values of *T* are higher or lower than all values of *R*, respectively. The resulting value is defined by:

$$AUC(T, R) = AUC - 0.5$$

with *AUC*(*T*, *R*) ∈ [-0.5, 0.5]. An *AUC*(*T*, *R*) of 0 indicates an equal distribution of datasets[33]. All remaining features are ranked in descending order of the absolute *AUC*(*T*, *R*). Additionally, for all remaining features, CPP compares *T* and *R* using the Mann-Whitney-U test and computes the Benjamini-Hochberg[34] adjusted *P* values, highly correlating with the absolute *AUC*(*T*, *R*).

4. **Feature filtering**: CPP conducts stepwise filtering of the ranked features in descending order, whereby features are compared with all features that have been ranked higher and not removed before. The highest-ranked feature is chosen without comparison. A feature f1, compared to a higher ranked feature f2, is removed if (1) the scales of f1 and f2 are in the same scale category, (2) the overlap of the residue positions of f1 and f2 is higher than a user-defined threshold named *max_overlap* and (3) the Pearson correlation coefficient between the scales is higher than a user-defined threshold called *max_cor*. For example, if the scales of the top five features are classified into different scale categories, they are all kept; next, if the 6th feature (1) has the same scale category as one of the first five features, (2) the residue positions of the 6th feature and the feature with the same scale category overlap by at least *max_overlap*%, and (3) their scale values correlates with a Pearson coefficient higher than *max_cor*, then this 6th feature would be removed. In this step, CPP reduces the number of features further down to a user-defined threshold of *n_filter*, for instance, from 5.000 to 100 for *n_filter*=100.

The output of CPP is a feature table comprising information about the features (e.g., the feature name or the scale category), the computed measures (e.g., the absolute *AUC*(*T*, *R*) or the *std*(*T*)), and the statistical significance (Supplementary Data 8).

In summary, CPP compares two sets of protein sequences (the test dataset and the reference dataset) to identify their most distinctive features in four steps: First, CPP creates all possible features for given parts, splits, and scales. Second, CPP removes features with a standard deviation for the test dataset higher than *max_std_test* and selects the top *pct_pre_filter* with the highest mean difference between test and reference dataset. Third, CPP ranks all remaining features in descending order of the absolute adjusted *AUC*. Last, CPP filters the remaining features for redundancy regarding scale categories, sequence positions (via *max_overlap*), and scale correlation (via *max_cor*). The last step is performed until a user-defined maximum of *n_filter* features remained.

Comparing SUBEXPERT (test set) against OTHERS (reference set), CPP created 131,670 features for 3 parts (TMD, JMD-N-TMD-N, and TMD-C-JMD-C), 330 splits (120 segments, 182 patterns, and 28 periodic patterns), and 133 min-max normalized scales (Set 5, see 'Selection of scales'), where parts and scales were optimized (see 'Benchmarking CPP without and with NONSUBPRED'). To efficiently pre-filter these features, *max_std_test*=0.2 and *pct_pre_filter*=0.05 were used, yielding 6,583 features. In the filtering

step, a value of 0.5 was empirically chosen for *max_overlap* and *max_cor* as a well-balanced trade-off between a too strict and a too permissive threshold (Supplementary Data 9). Overly strict settings (e.g., 0.25) decrease the number of significant features and can yield fewer features than selected by *n_filter*. Conversely, although too mild settings (e.g., 0.75) increase the number of significant features, they lead to redundancy regarding scales and sequence position. Sets of 150 and 100 features were tested in the subsequent steps. Unless specified otherwise, the 150-feature set will be used to illustrate the following steps, as it yielded best performance for the TMHMM annotation in dataset 1 (see 'Aggregation of prediction results', Supplementary Fig. 5d).

Substrate prediction by machine learning

Feature representation

Each protein was encoded by an n-dimensional numerical feature vector $\boldsymbol{x} = [x_1, \dots x_i, \dots, x_n]$, where x_i is the feature value for the *i*-th feature obtained by CPP and *n* is equal to *n_filter* (e.g., 150 features). A dataset with *m* proteins was encoded by a numerical $m \times n$ feature matrix:

$$\boldsymbol{X} = \begin{bmatrix} x_{11} & \cdots & x_{1n} \\ \vdots & x_{ji} & \vdots \\ x_{m1} & \cdots & x_{mn} \end{bmatrix} = \begin{bmatrix} \boldsymbol{x}_1 \\ \vdots \\ \boldsymbol{x}_m \end{bmatrix}$$

where *m* is the number of proteins with *j* indicating the *j*-th protein. Feature representation based on CPP only relies on the sequence parts of a protein. No further evolutionary or structural information, such as multiple sequence alignments or 3D structures, is needed.

Machine learning models

To predict γ-secretase substrates, 10 different machine learning classification model types were employed. We used 4 tree-based models (random forest, extremely randomized trees, xgboost, and catboost), 2 linear models (linear discriminant analysis and logistic regression) 1 kernel-based model (support vector machine) 1 neural network (multi-layer perceptron), and 2 ensemble models (a voting classifier and a stacking classifier). As base-models for both ensemble models, we used random forest, catboost, linear discriminant analysis, logistic regression, and support vector machine. For the stacking model, support vector machine was chosen as a meta-model or final estimator. All models, except xgboost and catboost, were implemented in scikit-learn. An overview of the machine learning model types including optimized hyperparameters is given in Supplementary Data 10. The default settings of the respective implementations were used if not specified otherwise. For benchmarking, support vector machine and random forest were employed as validation models, which are the most popular models for small datasets[35,36].

Evaluation measures

To evaluate the performance of machine learning models, six performance measures—accuracy (ACC), sensitivity or recall (REC), precision (PRE), F1 score (F1), true negative rate (TNR), and balanced accuracy (bACC)—were used, defined as follows:

$$\text{ACC} = (\text{TP} + \text{TN})/(\text{TP} + \text{TN} + \text{FP} + \text{FN})$$
$$\text{REC} = \text{TP}/(\text{TP} + \text{FN})$$
$$\text{PRE} = \text{TP}/(\text{TP} + \text{FP})$$
$$\text{F1} = 2\text{TP}/(2\text{TP} + \text{FP} + \text{FN}) = 2(\text{REC} * \text{PRE})/(\text{REC} + \text{PRE})$$
$$\text{TNR} = \text{TN}/(\text{TN} + \text{FN})$$

$$\text{bACC} = (\text{REC} + \text{SPC})/2 = (\text{TP}/(\text{TP} + \text{FN}) + \text{TN}/(\text{TN} + \text{FP}))/2$$

where TP, FP, TN, FN are the number of true positives, false positives, true negatives, and false negatives, respectively, and $\text{SPC} = \text{TN}/(\text{TN} + \text{FP})$ is the specificity. Cross-validation as implemented in sklearn.model_selection.cross_validation and leave-one-out cross-validation as implemented in sklearn.model_selection.LeaveOneOut were used for model evaluation.

Derivation of the optimal scale and part sets for CPP

To select the best (i.e., most informative and non-redundant) sets of scales and parts for CPP, we trained 20=5×4 support vector machine classification models. The CPP features for these models were obtained by combining 5 different sets of scales (Set 1–5, see 'Selection of scales') and the following 4 sets of parts: (1) TMD; (2) TMD-JMD; (3) TMD, JMD-N-TMD-N, TMD-C-JMD-C; and (4) TMD, TMD-E, JMD-N-TMD-N, TMD-C-JMD-C. All models were trained on SUBEXPERT (n=63) against NONSUB (n=14). Proteins were represented by a $m \times n$ feature matrix, where m=77=63+14 is the number of proteins and n=150 is the number of CPP features. Because of this small and imbalanced dataset, we used leave-one-out cross-validation with balanced accuracy for model evaluation.

We also trained 5 baseline support vector machine models, with features computed as the mean scale value over all TMD residues. Using scale sets 1–5, the feature matrix for these baseline models contained n=586, 232, 192, 161, 133 features, respectively. These models showed a low performance (50% balanced accuracy), whereas models using CPP features with different parts and non-redundant scale sets improved to around 80% balanced accuracy. The combination of scale set 5 (n=133) and part set 3 (TMD, JMD-N-TMD-N, TMD-C-JMD-C) resulted in the highest balanced accuracy of 84% (Supplementary Fig. 4g) and was therefore used for further analysis steps.

Computational non-substrate identification by dPULearn

To balance the initial training dataset of 63 substrates (SUBEXPERT) and 14 non-substrates (NONSUB), we employed ideas from positive-unlabeled (PU) learning[37]. PU learning is an increasingly popular subfield of machine learning[38,39] aiming at identifying negative samples from unlabeled samples. Common PU learning strategies usually involve two steps[37]. First, negative samples are identified that are most distant from positive samples using distance measures such as Euclidean distance. Second, this set of negatives is iteratively extended by (semi-)supervised learning models (e.g., support vector machine) that are trained using the current dataset of positive and negative samples to predict new negatives for subsequent training iterations[37,40,41]. However, the disadvantage of the supervised learning models used in step 2 is that they are mainly non-deterministic (i.e., stochastic), leading to irreproducible identification of negative samples (see 'Benchmarking dPULearn').

To tackle this problem, we developed a deterministic PU learning method, named dPULearn (Supplementary Fig. 3a). dPULearn is inspired by PU learning techniques of step 1 and follows the information-theoretic PU learning approach outlined in ref. [42]. To reduce potential bias caused by dominant features, dPULearn compresses the entire feature space by principal component analysis and iteratively identifies negatives (non-substrates) based on the most informative principal components (PCs), i.e., those with the highest explained variance.

Using all proteins from SUBEXPERT (n=63) and OTHERS (n=631) as positive and unlabeled samples, respectively, our aim was to extend NONSUB (n=14, negative samples) by n=49=63-14 additional non-substrates delineated from OTHERS (Supplementary Fig. 3c). To this end, the original k-dimensional feature space obtained by CPP (k=100 and 150 features were tested) was reduced via a principal component analysis. The first m=7 PCs were selected to cover a maximum of 80% total variance, an empirically optimized threshold (see 'Benchmarking dPULearn'). For each of the m components, $n_{PCi} = \lceil 49 \times p_i \rceil$ proteins were

selected, where p_i is the relative proportion of the explained variance of the i-th component, and $\lceil \ldots \rceil$ indicates rounding up. For example, the first component (PC1) accounts for 56.1% of the total variance and its relative proportion of explained variance is therefore $p_1 = 56.1/80 = 0.70$, yielding $n_{PC1} = \lceil 49 \times 0.70 \rceil = 35$ additional non-substrates to be selected (Supplementary Fig. 3d). Generally, the selection process of the dPULearn algorithm (Supplementary Fig. 3a) involves the following three steps for each of the m PCs:

1. Computing the average principal component value (mean PCi) of the positive samples (in our case, proteins in SUBEXPERT).
2. Determining the distance of each unlabeled sample (proteins in OTHERS) to mean PCi.
3. Selecting the n_{PCi} proteins that exhibit the greatest absolute distance to mean PCi to obtain reliable negative samples (additional non-substrates).

These steps are performed for all components in descending order of explained variance, where already selected proteins are not considered for subsequent components. Through this approach, dPULearn prioritizes PCs with higher explained variance by both their order during the selection process and the rounding procedure, which yields the maximum number of selected negatives for each component by rounding up. Thereby, PCs with low explained variance are excluded, decreasing the risk of false negatives (i.e., incorrectly recognizing true substrates as non-substrates).

For an optimized number of 150 CPP features (Supplementary Fig. 5d**,** dataset 1, TMHMM), dPULearn identified a set of 49 additional non-substrates, referred to as NONSUBPRED. These proteins are most distinct from the 63 substrates in SUBEXPERT regarding the first 7 PCs, covering 74.6% total variance. NONSUBPRED (n=49) extended NONSUB (n=14) to achieve a balance of substrates in SUBEXPERT (n=63) and non-substrates (n=63=49+14).

For the UniProt and Phobius annotation, 53 and 51 additional non-substrates were identified using 150 and 100 features, respectively. The NONSUBPRED sets for the different TMD annotations (Supplementary Data 11) showed only moderate overlap (Supplementary Fig. 3b), reflecting the inherent complexity in defining membrane boundaries.

Benchmarking dPULearn

We benchmarked dPULearn against a well-established PU learning framework, Elkanoto[40], as implemented in pulearn.ElkanotoPuClassifier, using random forest and support vector machine as supervised learning models. Three hyperparameter settings were tested for the random forest (number of trees=32, 64, 100) and support vector machine (regularization parameter (C)=0.1, 1, 10) models. Since reliable negatives could not be directly retrieved in this implementation, the ElkanotoPuClassifier was trained on positive and unlabeled data (SUBEXPERT and OTHERS, respectively), and the unlabeled samples with the lowest prediction scores were considered as reliably identified negative samples. Besides this stochastic approach, we tested an alternative deterministic method that identifies negative samples from unlabeled samples using the greatest Euclidean distance to the mean of the positive samples—step 1 of common two-step PU learning methods such as in ref. [41]. For dPULearn, we tested three values for the maximum total variance to be covered (70%, 80%, and 90%) by principal component analysis (PCA). The same set of CPP features (as described before, see 'Computational non-substrate identification by dPULearn') was used for all approaches.

All proteins of SUBEXPERT (n=63) and OTHERS (n=631) were used as positive and unlabeled samples, respectively. Given the set of known non-substrates (NONSUB, n=14), we used each method to identify n=49=63-14 additional non-substrates from OTHERS. To validate the identification methods, we employed random forest and support vector machine models for two different validation approaches. First, we performed leave-one-out cross-validation for

each identification method using the balanced datasets of all substrates (SUBEXPERT) and known non-substrates (NONSUB) including the set of identified non-substrates. Accuracy was used as a performance measure, which gives the same results as balanced accuracy if the dataset is balanced. Second, to test the generalizability of our models on an independent test set, the true negative rate (TNR) of all known non-substrates (NONSUB) was computed for the validation models trained on SUBEXPERT against the set of identified non-substrates for each identification method.

All models showed a good performance with an accuracy of around 90%, but the comparison of TNR values revealed substantial differences in their generalization ability (Supplementary Fig. 4a). The Elkanoto approaches with support vector machine showed higher accuracy values than all other approaches but worse TNR results, which might indicate overfitting. The Elkanoto approaches with random forest showed slightly lower accuracy results but the highest TNR of all approaches. Regarding the deterministic methods, the Euclidean distance-based method might suffer from overfitting indicated by a low TNR. In contrast, dPULearn showed a good performance for both measures in all PCA variance settings. Interestingly, an increase in the covered variance increased the TNR but decreased the ACC. Therefore, we chose dPULearn with a maximum of 80% total variance as the best deterministic method because it represents a good trade-off between accuracy and TNR. This model performed similarly or slightly worse than the best stochastic models (random forest with 64 or 100 trees).

Since not only the model performance but also the reproducibility of the results is a crucial benchmarking criterion, we quantified the reproducibility of dPULearn (with 80% variance) and two Elkanoto approaches (support vector machine with C=1, random forest with 100 trees) as follows. We ran each method 100 times and computed the relative maximum overlap of identified non-substrates, i.e., the percentage of non-substrates identified in each run relative to the total number of identified non-substrates. While dPULearn was 100% reproducible, the Elkanoto approaches using support vector machine and random forest showed only 14% and 0% reproducibility, respectively (Supplementary Fig. 4b). Next, we compared the impact of different parameter settings on the overlap between sets of identified non-substrates. The sets obtained by the support vector machine-based and random forest-based methods showed a moderate (27 out of a maximum of 49 proteins) and no overlap (Supplementary Fig. 4c, d), respectively. In contrast, the sets identified by dPULearn with three different variance settings (70%, 80%, and 90%) had an overlap of 41 proteins (Supplementary Fig. 4e). To benchmark dPULearn against random sampling, we used a one-sample t-test comparing the accuracy value retrieved using dPULearn (80% variance) against the accuracy values for 100 models trained on randomly sampled sets of identified non-substrates (Supplementary Fig. 4f), showing a significantly better ($P<0.001$) performance for dPULearn.

We conclude that the stochastic nature of the supervised learning models notably hampers the reproducibility of the non-substrate identification. Consequently, we chose dPULearn (80% maximum variance) to derive reliable non-substrate datasets, despite a certain dependence on the TMD annotation (Supplementary Fig. 3b, see above). See Supplementary Data 12 for benchmarking results of dPULearn predictions and reproducibility.

Benchmarking CPP without and with NONSUBPRED

We benchmarked CPP without and with the use of the 49 non-substrates (NONSUBPRED) identified by dPULearn. To evaluate CPP without NONSUBPRED (Supplementary Fig. 4g), we employed 5 support vector machine models without CPP features, referred to as baseline models, and 20=5x4 support vector machine models with CPP features. All models were trained on SUBEXPERT (n=63) against NONSUB (n=14), as explained in the 'Derivation of the optimal scale and part sets for CPP' section. For the baseline models, features were obtained for each scale by computing their average value over the entire TMD sequence. CPP

benchmarking was performed for all TMD annotations, but only the results for the TMHMM annotation are presented for the sake of clarity. The results demonstrate a general improvement from 50% balanced accuracy to over 80% when CPP features are utilized, best for scale set 5 and part set 3 (84%, bold square, Supplementary Fig. 4g).

Next, we assessed CPP with NONSUBPRED (Supplementary Fig. 4h) to test whether the balanced dataset leads to further improvement. We trained and evaluated 20 support vector machine and 5 additional baseline models, similar to the previous setup, but the models were trained on SUBEXPERT (n=63) against NONSUB (n=14) plus NONSUBPRED (n=49). Accuracy was chosen as performance measure instead of balanced accuracy because the datasets were balanced and therefore both measures yielded identical results. All 5 baseline models improved their performance from 50% balanced accuracy to around 70% accuracy, while models with redundancy-reduced scale sets and multiple parts could even improve from 80% balanced accuracy to about 90% accuracy. As before, the model using scale set 5 and part set 3 as input for CPP showed the highest performance, reaching 92% accuracy (bold squares, Supplementary Fig. 4g, h). See results for all TMD annotations in Supplementary Data 12.

Benchmarking CPP and dPULearn against deep learning-based embeddings

We benchmarked CPP and dPULearn against scale-based and deep learning-based feature engineering approaches. As an alternative to dPULearn, the Synthetic Minority Over-sampling Technique (SMOTE)[43,44] data augmentation framework was used for data expansion. As deep learning-based approach, protein embeddings (i.e., self-learned, rather than knowledge-based, scale-like properties) were used, applied via transfer learning[19,45]. For performance evaluation, we trained support vector machine models with features obtained by the different feature engineering approaches on SUBEXPERT (n=63) against NONSUB (n=14). Additionally, the NONSUB dataset was extended either by NONSUBPRED (n=49) identified via dPULearn or by 49 synthetic non-substrates artificially generated through SMOTE.

Three feature engineering approaches were compared: (a) CPP with the optimized sets of 3 parts (TMD, JMD-N-TMD-N, TMD-C-JMD-C) and 133 scales; (b) scale-based features, i.e., the average scale values over the entire TMD-JMD sequence using the same optimized scale set as in (a); and (c) embeddings obtained from the state-of-the-art protein deep language model ProtTrans5 (ProtT5)[46], which returns an $n \times 1024$ matrix for a sequence with n amino acids, where 1024 is the number of embeddings or dimensions. As for scale-based features, we computed the average value for each dimension over the entire TMD-JMD sequence. In addition, we tested embeddings either averaged over the entire sequence or compressed by principal component analysis, leading to similar or worse results.

One support vector machine model was trained for each combination of feature engineering method (scale-based, ProtT5, or CPP) and data expansion technique (no expansion ('None'), SMOTE, or dPULearn). To further optimize the performance of CPP and dPULearn, we evaluated different combinations of top CPP feature sets with an increasing number of features n (n=25, 50, 75, 100, 125, 150) used for model training and for non-substrate identification, yielding 36=6x6 evaluations (Fig. 3b). Validation was performed using leave-one-out cross-validation with balanced accuracy as an evaluation metric, only considering proteins from SUBEXPERT and NONSUB to prevent data leakage and maintain consistency. See benchmarking results of CPP with dPULearn against scale-based feature engineering and ProtT5 embeddings in Fig. 3a and Supplementary Data 12.

Training datasets

To address the inherent uncertainty in defining the membrane boundaries, our machine learning pipeline (Supplementary Fig. 5a) was performed separately for each dataset-annotation combination. Two training datasets (dataset 1 and dataset 2) were collated for each TMD annotation (UniProt, TMHMM, Phobius). Both datasets differed in their composition of used

substrates: dataset 1 included only expert-curated substrates (SUBEXPERT), while dataset 2 expanded this group by a literature-based substrate set (SUBLIT). As shown for the TMHMM annotation (Supplementary Fig. 5b), dataset 1 was balanced containing 63 substrates (SUBEXPERT) and 63 non-substrates (14 from NONSUB and 49 from NONSUBPRED identified by dPULearn), while dataset 2 comprised 136 substrates (63 from SUBEXPERT and 73 from SUBLIT) and the same 63 non-substrates as in dataset 1. This ensures comparability by prioritizing consistency between datasets, rather than focusing solely on sample size ratio.

Learning strategy

To improve the reproducibility of our machine learning pipeline, we performed 25 independent training rounds (Supplementary Fig. 5a), yielding a Monte Carlo estimate of prediction scores[47]. In each training round, a dataset was randomly split into a training set (80%) and a test set (20%), both containing a balanced proportion of substrates and non-substrates. As recommended for small sample sizes[36], we used a nested cross-validation approach, where the training set was used for feature selection and hyperparameter optimization by a 5-fold cross-validation. The test set was then used for an independent evaluation of the optimized models at the end of each round.

Feature selection

For feature selection (Supplementary Fig. 5a), we first removed highly correlated features from a given feature matrix (see 'Feature representation') if the Pearson correlation of a feature with a higher ranked feature exceeded a specific threshold. In this feature pre-selection step, we tested a combination of the top 150 or top 100 features with Pearson correlation thresholds of 0.8, 0.85, or 0.9—yielding 6=2×3 possible combinations (Supplementary Fig. 5c). Next, for each combination, a stepwise feature elimination algorithm was applied. This involved training a random forest model, obtaining the importance of each feature[48], evaluating the model performance using a 5-fold cross-validation with F1 score (see 'Evaluation measures'), and ultimately removing all features with the lowest importance. This elimination process was repeated until only a minimum of 25 features remained. The feature set of the random forest model with the highest F1 score was selected, whereby only models with less than 50 features were considered.

Model optimization and evaluation

We optimized the hyperparameters of the 8 non-ensemble machine learning model types (random forest, extremely randomized trees, xgboost, catboost, linear discriminant analysis, logistic regression, support vector machine, multi-layer perceptron) using grid search, as implemented in sklearn.model_selection.GridSearchCV. For validation, we used a 5-fold cross-validation with accuracy as the evaluation measure. The optimized models were then used for 2 further ensemble models (see 'Machine learning models').

To assess the performance of the 10 models in each training round (Supplementary Fig. 5a), we computed the average accuracy over the 5 folds, referred to as 'cv_mean'. In addition, the performance of each model on the independent test set was evaluated using ACC, REC, PRE, and the F1 score (see 'Evaluation measures'). To determine the overall model performance across all 25 rounds, we calculated the average of each metric and each model. Finally, each of the 250=25×10 models predicted the substrate probability of a given sequence, and the mean of these predictions was computed, referred to as the dataset-annotation-specific 'substrate prediction score'.

Aggregation of prediction results

To reduce the impact of potential selection bias due to differences in dataset composition or TMD annotation, we selected and aggregated the best approaches for each dataset-annotation

combination. This comprised in total 6=2×3 training approaches, corresponding to 2 datasets (Supplementary Fig. 5b) and 3 TMD annotations (UniProt, TMHMM, Phobius). Each dataset-annotation combination was optimized for 6 feature pre-selection setups (Supplementary Fig. 5c). We compared 36=6×6 training approaches—each comprising 250 trained models (see 'Model optimization and evaluation')—and selected the best approach for each dataset-annotation combination based on the average accuracy (Supplementary Fig. 5d). This yielded the 6 best approaches, from each of which we derived the average prediction score, a dataset-annotation-specific substrate prediction score. Finally, the 6 best approaches were aggregated by averaging their prediction score (1500=6×250 trained models, Supplementary Fig. 5e). This score was used as the final 'substrate prediction score' and its standard deviation was computed over scores of the 6 best approaches (Fig. 4d–f, Supplementary Data 13).

To select the best dataset-annotation combination for subsequent steps, we compared the evaluation results of the 6 best approaches (Supplementary Fig. 5d, f). Training on dataset 1 with TMHMM annotation yielded the highest accuracy of 96% and is therefore used in subsequent steps if not stated otherwise. Additionally, the impact of the dataset was compared by aggregating over the 3 TMD annotations (750 models, Supplementary Fig. 6a). See Supplementary Data 13 for the prediction results for all 3,782 single-span TPs with a minimum JMD length of 10 amino acids (2,003 from human, 1,765 from mouse, 12 from rat, and 2 from chicken).

Confidence-based substrate classes

Based on the substrate prediction score [0–100%], we classified single-span TPs into the following four substrate classes, distinguished by varying prediction confidence:

- **HC substrate**: High-confidence substrate, prediction score >=80%.
- **LC substrate**: Low-confidence substrate, prediction score >=50% and <80%.
- **LC non-substrate**: Low-confidence non-substrate, prediction score <50% and >20%.
- **HC non-substrate**: High-confidence non-substrate, prediction score <= 20%.

Applying these confidence-based classes to the human N-out proteome (n=1534) yielded 250 HC substrates, 587 LC substrates, 599 LC non-substrates, and 98 HC non-substrates (Fig. 4b). This classification is solely based on protein sequence features described above and does not take into account molecular biological criteria for bona fide γ-secretase substrates, such as co-expression and co-localization of the substrate with the γ-secretase or the presence of a short ectodomain, either naturally or due to shedding prior to γ-secretase cleavage (see 'Datasets').

Explainable AI

Explainable AI using SHAP

Explainable artificial intelligence (explainable AI) is a machine learning subfield that aims to make the results of machine learning models interpretable. Within this field, the game theoretic SHapley Additive exPlanations (SHAP)[49,50] framework is the state-of-the-art approach for explaining prediction scores of individual samples. SHAP provides an additive model of feature contributions based on the sum of Shapley values (SHAP values) and a model-dependent 'base value'. Four key concepts of the SHAP framework are defined as follows:

- **Feature impact**: Positive or negative contribution of a feature, resulting in the model output for a sample (i.e., prediction score expressed by SHAP values) to be higher or lower, respectively.

- **Base value**: Average model output of SHAP values over the entire training dataset of m samples and n features defined as: $B = \frac{1}{mn} \sum_i^m \sum_j^n S_{i,j}$, where $S_{i,j}$ is the SHAP value for the i-th sample and the j-th features. In case of a balanced training dataset, B is around 0.5.
- **SHAP output**: Sample-specific sum of the base value defined above and all respective feature impacts. For a given sample and n features, its SHAP output O is defined as: $O = B + \sum_j^n S_j$, where S_j is the SHAP value of the j-th feature. The SHAP output approximates the prediction score of the given sample[50].
- **Feature importance**: The absolute value of the feature impact, used for feature ranking.

The additive approximation of the prediction score by the SHAP output can be explicitly visualized through SHAP force plots (Supplementary Fig. 9b).

Combining CPP with SHAP

To obtain the feature impact of individual proteins for a dataset-annotation-specific machine learning approach, we computed SHAP values for the best feature set and the best tree-based models (random forest, extremely randomized trees, xgboost, catboost) of each training round (Supplementary Fig. 9a). Since SHAP values depend on the training dataset, each model was trained on the total training dataset (either dataset 1 or dataset 2) to keep the retrieved models and features consistent. We then utilized the tree-based explainer as implemented in shap.TreeExplainer (with 'probability' model output) to compute SHAP values, which are thus provided as probabilities that sum up to the model´s probability output[49,50]. The tree-based explainer was favored over the linear or kernel-based explainers due to its faster computation of SHAP values[49] and its probability output, which enables the normalization of SHAP values, facilitating the aggregation across multiple models.

For each sample and feature obtained by CPP (150 features), the average SHAP values were computed over the 4 tree-based models and 25 training rounds. If a feature was removed during feature selection, its SHAP value was set to zero for all samples.

Consider a $m \times n \times k \times l$ matrix of SHAP values (Supplementary Fig. 9a), where m=126 is the number of samples (proteins in dataset 1), n is the number of all features obtained by CPP, k=25 is the number of training rounds, and l=4 is the number of models trained per round. We computed the average SHAP value for each feature and sample over all rounds and models—as a result, the initial matrix was aggregated to a $m \times n$ matrix. Given k=25 and l=4 defined as before, a $k \times l$ matrix of base values was aggregated to then compute the average base value across this entire matrix.

To compute the feature impact for an individual sample, we normalized its average SHAP values, represented by the array $S = [S_1, \dots, S_i]$ containing i SHAP values. We applied to each SHAP value in S the function $f(s) = s/sum(abs\_array(S))$, where $abs\_array(S)$ yields an array of absolute SHAP values. This results in a new array $S_{impact} = [f(s_1), \dots, f(s_i)]$, where each element is the normalized impact of its corresponding feature. Ideally, the sum of these normalized feature impacts (the SHAP output) should be equal to the prediction score.

To compute the feature importance (i.e., the absolute feature impact) for an individual sample, we applied $g(s) = abs(s)/sum(abs\_array(S))$ to S instead of $f(s)$, where $abs(s)$ is the absolute value of the SHAP value s. Next, the average feature importance for all training samples was obtained by computing the average of the absolute SHAP values. Consider a $m \times n \times k \times l$ matrix of absolute SHAP values, where m=126, n=150, k=25, and l=4 are defined as before. The importance of each of the n features is the average absolute SHAP value over all 126 samples, 25 training rounds, and 4 tree-based models. The average feature importance was used to rank features in descending order.

CPP-SHAP plots

By combining the CPP feature concept with the additive SHAP feature model, we can reveal the residue-specific impact of each feature for individual protein sequences. This approach extends the SHAP framework to sequence-based protein prediction tasks, enabling positional interpretation[51] with incorporation of physicochemical properties. To effectively depict these insights, we developed four distinct visualizations: the 'CPP-SHAP ranking plot', 'CPP-SHAP profile', 'CPP heatmap', and 'CPP-SHAP heatmap' (Supplementary Fig. 9a). The first two visualizations can not only be applied to individual proteins but also to a group of proteins (typically the CPP test set, such as SUBEXPERT), then referred to as 'CPP ranking plot' and 'CPP profile'.

The CPP-SHAP ranking plot (e.g., Fig. 7a) shows the top *n*-ranked features, 15 by default, defined as a scale-part-split combination (see 'Idea of the CPP algorithm'). The difference between the feature value of a selected protein and the mean of the reference set (e.g., OTHERS) is given as a positive/negative bar chart. Additionally, the positive and negative impact of each feature is depicted. At a group level, the CPP ranking plot shows the mean difference for a test set (e.g., SUBEXPERT) as well as the feature importance instead of the feature impact (e.g., Supplementary Fig. 9c).

The CPP-SHAP profile (e.g., Fig. 7b) shows the cumulative feature impact per residue position as a positive/negative bar chart for a selected protein. Each feature's negative or positive impact (blue or red, respectively) is assigned to a specific residue and normalized position-wise and by the sum of their absolute values. At a group level, the CPP profile shows the cumulative feature importance, which is undirected, instead of the cumulative feature impact (e.g., Fig. 2b), which is either positive or negative (i.e., directed).

The CPP heatmap (e.g., Fig. 7c) shows the feature value differences (as described for CPP ranking plots) per residue position and scale subcategory. The classification of scale subcategories (see 'Selection of scales') is indicated by a defined color code. Instead of the mean difference, the CPP-SHAP heatmap (e.g., Fig. 7d) shows the feature impact per residue position and scale subcategory for the selected protein. Subcategories are indicated as for CPP heatmaps. At a group level, the CPP heatmap is part of the 'CPP feature map' (Fig. 2c), which additionally integrates the feature importance per residue position and scale subcategory.

Fuzzy labeling

We developed a 'fuzzy labeling' approach to determine the feature impact for any given input protein sequence not contained in an initial training dataset. First, the prediction score for the protein (ranging from 0% to 100%) is derived using tree-based machine learning models, trained as described before (see 'Combining CPP with SHAP', 4 models trained over 25 rounds). Next, the protein of interest is added to the training dataset. The models are then re-trained over 25 rounds, labeling the protein as positive (substrate) or negative (non-substrate) corresponding to its prediction score. For example, a protein with a prediction score of 60% will be labeled as a substrate in 60% of the rounds (15 rounds) and as a non-substrate in the remaining 40% (10 rounds), aiming to approximate the 60% prediction score by the sum of feature impacts (SHAP output) of around 0.6. We refer to this procedure as 'fuzzy labeling' because samples are neither only labeled as a substrate nor as a non-substrate. This approach allowed for the best approximation of the prediction score by the SHAP output.

Fuzzy labeling was applied to TREM2 (Supplementary Fig. 10b–d), an important substrate of γ-secretase contained in SUBLIT. For dataset 1, tree-based models were re-trained for 25 rounds using m=127=126+1 samples, including TREM2. With a prediction score of 22%, TREM2 was labeled 6=[25×0.22] times as a substrate, resulting in a SHAP output of 0.27. In contrast, with dataset 2, TREM2 was labeled as a substrate in all 25 rounds, resulting in a SHAP output of 0.77. Due to the imbalanced nature of dataset 2, comprising 139 substrates and 63 non-substrates, the base value was 0.65 instead of 0.5. Adjusting the SHAP output to a base

value of 0.5 leads to a SHAP output of 0.62=0.77-0.15, corresponding to the prediction score of 62%. See Supplementary Data 15 for a detailed CPP-SHAP analysis (i.e., obtaining and visualizing feature impact for an individual protein) for APP, NOTCH2, and ITGB1.

Clustering based on feature impact
We clustered substrates and non-substrates based on the Pearson correlation of their feature impacts. The hierarchical agglomerative clustering algorithm with complete linkage was used as implemented in scipy.cluster.hierarchy.linakge. Given dataset 1 with TMHMM annotation, five clusters were identified. The Pearson correlation matrix of the clustered dataset was visualized using seaborn.clustermap (Fig. 8a). See detailed results of clustering in Supplementary Data 16.

Functional bioinformatics analysis of γ-secretase substrates

Dataset of human N-out proteome
For a functional analysis (Fig. 9a), we considered substrate predictions for the entire human N-out proteome (n=1534), i.e., type I and type III single-span TPs. Based on our confidence-based substrate classification, the human N-out proteome comprises 98 HC non-substrates, 599 LC non-substrates, 587 LC substrates, and 250 HC substrates (Fig. 4b). 160 HC substrates have an unknown substrate status, referred to as 'new HC substrates'. In comparison, the remaining 90 HC substrates were already known, 59 from SUBEXPERT and 31 from SUBLIT (Fig. 9b). See datasets and results of functional analysis in Supplementary Data 17.

Enrichment analysis
To gain insights into potential functions of the HC substrates, we performed a gene ontology (GO)[52] and pathway enrichment analysis[53] (Fig. 9a). First, an enrichment analysis of GO terms for biological process (GO-BP), cellular component (GO-CC), and molecular function (GO-MF) was performed using the g:Profiler web server[54]. All HC substrates (n=250) were tested against the background of the whole human N-out proteome (n=1534; Supplementary Data 13). As recommended in ref. [53], a Benjamini-Hochberg FDR threshold of 0.05 and a term size restriction of 5–500 genes per term was used. The term list derived for each GO domain was summarized using semantic clustering by REVIGO[55] with a similarity cut-off of 0.5 and default settings. For each cluster, the enrichment score was computed by averaging the -log10 *P* values over all cluster members.

To characterize the differences between known substrates (SUBEXPERT and SUBLIT; n=147) and new HC substrates (n=160), a pathway enrichment analysis was performed utilizing the g:Profiler web server (with default background gene list) for the human KEGG, Reactome, and WikiPathways pathway databases.

Finally, the list of significant terms from the three pathway databases was clustered and visualized using Cytoscape (version 3.9.1)[56] and EnrichmentMap[57], with the edge similarity cut-off of 0.5 and the node significance cut-off of 0.05 q-value. Clusters were automatically named by the Cytoscape plugin AutoAnnotate using its MCL clustering algorithm and manually improved for biological consistency, resulting in 7 clusters. See Supplementary Data 18 for the g:Profiler output, including gene lists for each term, and see Supplementary Data 19 for the clustering results of REVIGO and EnrichmentMap.

Network analysis
A network analysis was performed (Fig. 9a) to analyze the role of HC substrates in functional protein networks. First, protein modules for the new HC substrates (n=160) were identified using the DOMINO web server[58] with the full STRING[59] network, yielding 8 modules. These

modules, including additional subnetwork members, were visualized using Cytoscape[56]. Next, we integrated the 8 modules with clustered pathway terms. For visualization purposes, we selected per module the two proteins with the highest number of pathway associations, prioritizing proteins experimentally validated in our study.

Subsequently, a whole network analysis was conducted for the N-out proteome on the STRING network obtained by the Cytoscape StringApp[60] (confidence>=0.4, default; 0.8 was also tested). Nodes represented genes, and edges represented functional interactions comprising direct (physical) or indirect (functional) associations[60]. Three network topology parameters were computed for each gene (node) using the Cytoscape NetworkAnalyzer[61]: (a) degree, defined as the number of functional associations (edges), (b) neighborhood connectivity, defined as the average degree of functionally associated genes (neighboring nodes), and (c) stress centrality, defined as the number of shortest paths running through the gene, where the shortest path is the minimum number of edges connecting two genes[60]. The differences between HC substrates and the other substrate classes were tested by two-sided Mann-Whitney-U test with Bonferroni correction. See Supplementary Data 17 for results of network parameters.

Analysis of pathway, disease, and mutation links

To gain insights into the biological functions of proteins within the human N-out proteome (n=1534), we analyzed protein links (i.e., protein associations based on knowledge from publicly available databases) with pathways, diseases, and mutations. For pathway links, we downloaded the complete Reactome database from the g:Profiler web server. To obtain disease links, we downloaded the complete DisGeNET[62] human disease-gene association database and retrieved all gene-disease associations from its 'geneDiseaseNetwork' table (n=3,261,324). DisGeNET provides a confidence score ranging from 0 to 1 (lowest to highest level of evidence, respectively), considering other curated sources like UniProt or GWAS studies. Only non-redundant links with a confidence score>=0.1 were kept (n=329,923). Mutation links were downloaded from the UniProt database[1] using its 'Natural variant' information. We kept only mutations with reported single nucleotide polymorphism within the TMD-JMD sequence, of which several are disease-associated.

For each protein of the human N-out proteome, the number of pathway, disease, and mutation links was retrieved (a total of 7625, 9457, 490, respectively; Supplementary Fig. 15a). The differences between HC substrates and the other substrate classes were tested by two-sided Mann-Whitney-U test with Bonferroni correction. See Supplementary Data 17 for pathway, disease, and mutation links of the complete human N-out proteome.

Computation of relevance score

To identify physiologically relevant new HC substrates, we computed a 'relevance score'. To this end, we first obtained 'new links' (Supplementary Fig. 15a, Supplementary Data 20) with pathways and diseases that were previously not associated with γ-secretase—i.e., its subunits nicastrin, PEN-2, presenilin (PS1 or PS2), and APH-1 (APH-1a or APH-1b)—or its known substrates (i.e., proteins from SUBEXPERT and SUBLIT). The relevance score comprised five factors: (a) existence of a 'new pathway link'; (b) existence of a 'new disease link'; (c) existence of mutations within the TMD-JMD sequence ('mutated TMD-JMD'); (d) whether the protein family to which the respective protein belonged was not contained in protein families of the known substrates ('new protein family'); and (e) whether the TMD-JMD sequence did not exhibit more than 30% sequence identity to any substrate from SUBEXPERT ('dissimilar TMD-JMD'; see next section for computation). Each relevance factor was assigned a value of 1 or 0 (true or false), and the relevance score was computed as their average (Fig. 4g, Supplementary Data 17). The 'new protein family' factor was determined by a simple heuristic: a new HC substrate was considered to belong to a new protein family if the first three letters of its gene name did not match with the gene name of any known substrate. The number of new

disease/pathway links for the 20 HC substrates with the most links was visualized by a chord diagram (Fig. 10c) using the mne_connectivity.viz.plot_connectivity_circle function.

Comparison of CPP with a similarity-based approach

For each protein from the human N-out proteome, we assessed its similarity with the closest (i.e., most similar) substrate from SUBEXPERT regarding the whole sequence and the TMD-JMD region using the BLAST algorithm[63]. Additionally, to incorporate the top 100 CPP features as a similarity measure, we calculated the pairwise Pearson correlation between the feature values of each protein in the human N-out proteome and substrates of SUBEXPERT. We then obtained for each protein its correlation with the closest (i.e., most highly correlated) substrate from SUBEXPERT, referred to as 'CPP feature correlation'.

For each of these three similarity measures, we assessed the difference between the HC substrate class and the remaining three substrate classes using the two-sided Mann-Whitney-U test with Bonferroni correction. To further compare the discriminatory power of the TMD-JMD sequence similarity and the CPP feature correlation, we min-max normalized both similarity measures on the human N-out proteome and compared their differences between HC non-substrates and HC substrates (Fig. 6h). See the results of the similarity measures for the complete human N-out proteome in Supplementary Data 17.

Co-expression analysis

We obtained RNA expression data from the Human Protein Atlas database (version 21.1) at the tissue and single-cell levels. The expression for γ-secretase was determined as the highest expression among the two homologs of its catalytic subunit, presenilin (PS1 and PS2), which are encoded by the PSEN1 and PSEN2 genes, respectively. To evaluate the co-expression relationship between γ-secretase and the four confidence-based substrate classes (see 'Confidence-based substrate classes'), we calculated the Pearson correlation between the expression values of γ-secretase and each protein in the human N-out proteome. The differences between HC substrates and the other substrate classes were tested by a two-sided Mann-Whitney-U test with Bonferroni correction.

To reflect the expression levels of the two main sheddase families, β- and α-secretase, we considered the highest expression among BACE1 and BACE2, the only two β-secretase family members, and ADAM10 and ADAM17, the α-secretase proteases with the largest number of known substrates (Supplementary Data 3). See results of co-expression analysis in Supplementary Data 17 and expression data for human N-out proteins in Supplementary Data 21.

Supplementary Algorithms

Algorithm 1. Simple rounding procedure for splitting sequence parts into segments
If the part length L is not divisible by n, we used a simple rounding procedure to obtain segments of approximately equal length:

1. Compute the step-size $s = L/n$.
2. Calculate the non-rounded start and end position of the *i*-th segment as:

$$i_{start\_non-rounded} = (i-1) \times s$$
$$i_{end\_non-rounded} = i \times s$$

3. Determine the actual residue start and end position of the *i*-th segment as:

$$i_{start} = \lfloor i_{start\_non-rounded} \rfloor + 1$$
$$i_{end} = \lfloor i_{end\_non-rounded} \rfloor$$

where $\lfloor x \rfloor$ represents the floor function, rounding *x* down to the nearest integer (e.g., 2.9 to 2). Adding 1 to the start position ensures that the first segment begins at position 1 and the end position of one segment does not overlap with the start position of the next.

For example, splitting a sequence part of length *L*=21 into 4 segments (*n*=4), yields a step-size of $s = 5.25$ and the following segments:

- Segment(1,4): 5 residues (positions: {1,2,3,4,5}; non-rounded end position: 5.25)
- Segment(2,4): 5 residues (positions: {6,7,8,9,10}; non-rounded end position: 10.5)
- Segment(3,4): 5 residues (positions: {11,12,13,14,15}; non-rounded end position: 15.75)
- Segment(4,4): 6 residues (positions: {16,17,18,19,20,21}; non-rounded end position: 21)

Technical note: step 3 is achieved by Python´s float-to-int conversion and list indexing.

Algorithm 2. Scale selection based on subcategory coverage using AAclust
AAclust obtains a redundancy-reduced subset of a given scale set by optimizing the number of clusters *k* for a given *k*-based clustering model, such as agglomerative clustering. *k* is equivalent to the number of returned scales because AAclust selects for each cluster as a representative scale the *medoid* of the respective cluster—i.e., the scale with the highest correlation to the mean scale (called *centroid*) of a cluster. To optimize *k*, we obtained the number of all subcategories *n_subcat* within a given set of scales and iteratively increased *k* by 1 starting from *k*=*n_subcat*. In each step, the subcategory coverage *sc* was computed as follows:

$$sc = \frac{|\{subcat_s \mid subcat_s \in SUBCAT, s \in SCALE\}|}{n\_subcat},$$

where $subcat_s$ is the subcategory of a scale *s*, *SCALE* is a given set of scales, *SUBCAT* is the set of all subcategories to which scales in *SCALE* are assigned to, and $n\_subcat = |SUBCAT|$. *k* was increased until a complete subcategory coverage (i.e., *sc*=1) was reached. For example, consider Set 5 containing 353 scales assigned to 42 different subcategories, *SCALES* and *SUBCAT* contain all 353 scales and 42 subcategories, respectively. The first round of clustering starts with *k*=42, resulting in a redundancy-reduced set of 42 representative scales. Assume further that these 42 scales are assigned to 21 different subcategories, the subcategory coverage is then computed by *sc*=21/42=0.5. Now, k is increased until *sc*=1, which was achieved for *k*=133, yielding a redundancy-reduced set of 133 scales.

Supplementary References

1 UniProt Consortium, UniProt: the universal protein knowledgebase in 2023. *Nucleic Acids Res.* **51**, D523-D531 (2023).

2 Krogh, A., Larsson, B., von Heijne, G. & Sonnhammer, E. L. Predicting transmembrane protein topology with a hidden Markov model: application to complete genomes. *J. Mol. Biol.* **305**, 567-580 (2001).

3 Käll, L., Krogh, A. & Sonnhammer, E. L. L. Advantages of combined transmembrane topology and signal peptide prediction--the Phobius web server. *Nucleic Acids Res.* **35**, W429-W432 (2007).

4 Killian, J. A. & von Heijne, G. How proteins adapt to a membrane-water interface. *Trends Biochem. Sci.* **25**, 429-434 (2000).

5 Güner, G. & Lichtenthaler, S. F. The substrate repertoire of γ-secretase/presenilin. *Semin. Cell Dev. Biol.* **105**, 27-42 (2020).

6 Lyu, J., Yamamoto, V. & Lu, W. Cleavage of the Wnt receptor Ryk regulates neuronal differentiation during cortical neurogenesis. *Dev. Cell* **15**, 773-780 (2008).

7 Tousseyn, T., Thathiah, A., Jorissen, E., Raemaekers, T., Konietzko, U., Reiss, K., Maes, E., Snellinx, A., Serneels, L., Nyabi, O., Annaert, W., Saftig, P., Hartmann, D. & De Strooper, B. ADAM10, the rate-limiting protease of regulated intramembrane proteolysis of Notch and other proteins, is processed by ADAMS-9, ADAMS-15, and the γ-secretase. *J. Biol. Chem.* **284**, 11738-11747 (2009).

8 Vevea, J. D., Kusick, G. F., Courtney, K. C., Chen, E., Watanabe, S. & Chapman, E. R. Synaptotagmin 7 is targeted to the axonal plasma membrane through γ-secretase processing to promote synaptic vesicle docking in mouse hippocampal neurons. *Elife* **10**, e67261 (2021).

9 Hemming, M. L., Elias, J. E., Gygi, S. P. & Selkoe, D. J. Proteomic profiling of γ-secretase substrates and mapping of substrate requirements. *PLoS Biol.* **6**, e257 (2008).

10 Stützer, I., Selevsek, N., Esterhazy, D., Schmidt, A., Aebersold, R. & Stoffel, M. Systematic proteomic analysis identifies β-site amyloid precursor protein cleaving enzyme 2 and 1 (BACE2 and BACE1) substrates in pancreatic β-cells. *J. Biol. Chem.* **288**, 10536-10547 (2013).

11 Merilahti, J. A. M., Ojala, V. K., Knittle, A. M., Pulliainen, A. T. & Elenius, K. Genome-wide screen of γ-secretase-mediated intramembrane cleavage of receptor tyrosine kinases. *Mol. Biol. Cell* **28**, 3123-3131 (2017).

12 Li, W., Fu, L., Niu, B., Wu, S. & Wooley, J. Ultrafast clustering algorithms for metagenomic sequence analysis. *Brief. Bioinform.* **13**, 656-668 (2012).

13 Aßfalg, M., Güner, G., Müller, S. A., Breimann, S., Langosch, D., Muhle-Goll, C., Frishman, D., Steiner, H. & Lichtenthaler, S. F. Cleavage efficiency of the intramembrane protease γ-secretase is reduced by the palmitoylation of a substrate's transmembrane domain. *FASEB J.* **38**, e23442 (2024).

14 Wunderlich, P., Glebov, K., Kemmerling, N., Tien, N. T., Neumann, H. & Walter, J. Sequential proteolytic processing of the triggering receptor expressed on myeloid cells-2 (TREM2) protein by ectodomain shedding and γ-secretase-dependent intramembranous cleavage. *J. Biol. Chem.* **288**, 33027-33036 (2013).

15 Crooks, G. E., Hon, G., Chandonia, J. M. & Brenner, S. E. WebLogo: a sequence logo generator. *Genome Res.* **14**, 1188-1190 (2004).

16 You, Z. H., Chan, K. C. & Hu, P. Predicting protein-protein interactions from primary protein sequences using a novel multi-scale local feature representation scheme and the random forest. *PLoS One* **10**, e0125811 (2015).

17 Lee, I. & Nam, H. Sequence-based prediction of protein binding regions and drug-target interactions. *J. Cheminform.* **14**, 5 (2022).

18 Asgari, E. & Mofrad, M. R. Continuous Distributed Representation of Biological Sequences for Deep Proteomics and Genomics. *PLoS One* **10**, e0141287 (2015).
19 Eraslan, G., Avsec, Z., Gagneur, J. & Theis, F. J. Deep learning: new computational modelling techniques for genomics. *Nat. Rev. Genet.* **20**, 389-403 (2019).
20 Gosiewska, A., Kozak, A. & Biecek, P. Simpler is better: lifting interpretability-performance trade-off via automated feature engineering. *Decis. Support Syst.* **150**, 113556 (2021).
21 Greener, J. G., Kandathil, S. M., Moffat, L. & Jones, D. T. A guide to machine learning for biologists. *Nat. Rev. Mol. Cell Biol.* **23**, 40-55 (2022).
22 Quint, S., Widmaier, S., Minde, D., Hornburg, D., Langosch, D. & Scharnagl, C. Residue-specific side-chain packing determines the backbone dynamics of transmembrane model helices. *Biophys. J.* **99**, 2541-2549 (2010).
23 Kawashima, S., Pokarowski, P., Pokarowska, M., Kolinski, A., Katayama, T. & Kanehisa, M. AAindex: amino acid index database, progress report 2008. *Nucleic Acids Res.* **36**, D202-205 (2008).
24 Lins, L., Thomas, A. & Brasseur, R. Analysis of accessible surface of residues in proteins. *Protein Sci.* **12**, 1406-1417 (2003).
25 Koehler, J., Woetzel, N., Staritzbichler, R., Sanders, C. R. & Meiler, J. A unified hydrophobicity scale for multispan membrane proteins. *Proteins* **76**, 13-29 (2009).
26 Savojardo, C., Manfredi, M., Martelli, P. L. & Casadio, R. Solvent accessibility of residues undergoing pathogenic variations in humans: from protein structures to protein sequences. *Front. Mol. Biosci.* **7**, 626363 (2020).
27 Eyal, E., Najmanovich, R., McConkey, B. J., Edelman, M. & Sobolev, V. Importance of solvent accessibility and contact surfaces in modeling side-chain conformations in proteins. *J. Comput. Chem.* **25**, 712-724 (2004).
28 MacCallum, J. L. & Tieleman, D. P. Hydrophobicity scales: a thermodynamic looking glass into lipid-protein interactions. *Trends Biochem. Sci.* **36**, 653-662 (2011).
29 Breimann, S., Kamp, F., Steiner, H. & Frishman, D. AAontology: an ontology of amino acid scales for interpretable machine learning. *J. Mol. Biol.* **436**, 168717 (2024).
30 Ward, J. H. Hierarchical grouping to optimize an objective function. *J. Am. Stat. Assoc.* **58**, 236-244 (1963).
31 Breimann, S. & Frishman, D. AAclust: *k*-optimized clustering for selecting redundancy-reduced sets of amino acid scales. *Bioinform. Adv.* **4**, vbae165 (2024).
32 Mason, S. J. & Graham, N. E. Areas beneath the relative operating characteristics (ROC) and relative operating levels. *Q. J. R. Meteorol. Soc.* **128**, 2145–2166 (2002).
33 Hanley, J. A. & McNeil, B. J. The meaning and use of the area under a receiver operating characteristic (ROC) curve. *Radiology* **143**, 29-36 (1982).
34 Benjamini, Y. & Hochberg, Y. Controlling the false discovery rate: a practical and powerful approach to multiple testing. *J. R. Statist. Soc. B* **57**, 289-300 (1995).
35 Kokol, P., Kokol, M. & Zagoranski, S. Machine learning on small size samples: a synthetic knowledge synthesis. *Sci. Prog.* **105**, 368504211029777 (2022).
36 Vabalas, A., Gowen, E., Poliakoff, E. & Casson, A. J. Machine learning algorithm validation with a limited sample size. *PLoS One* **14**, e0224365 (2019).
37 Bekker, J. & Davis, J. Learning from positive and unlabeled data: a survey. *Mach. Learn.* **109**, 719-760 (2020).
38 Li, F., Wang, Y., Li, C., Marquez-Lago, T. T., Leier, A., Rawlings, N. D., Haffari, G., Revote, J., Akutsu, T., Chou, K. C., Purcell, A. W., Pike, R. N., Webb, G. I., Ian Smith, A., Lithgow, T., Daly, R. J., Whisstock, J. C. & Song, J. Twenty years of bioinformatics research for protease-specific substrate and cleavage site prediction: a comprehensive revisit and benchmarking of existing methods. *Brief. Bioinform.* **20**, 2150-2166 (2019).

39 Li, F., Dong, S., Leier, A., Han, M., Guo, X., Xu, J., Wang, X., Pan, S., Jia, C., Zhang, Y., Webb, G. I., Coin, L. J. M., Li, C. & Song, J. Positive-unlabeled learning in bioinformatics and computational biology: a brief review. *Brief. Bioinform.* **23**, bbab461 (2022).

40 Elkan, E. & Noto, K. Learning classifiers from only positive and unlabeled data. *Proc. ACM SIGKDD Int. Conf. Knowl. Discov. Data Min.*, 213-220 (2008).

41 Nan, X., Bao, L., Zhao, X., Zhao, X., Sangaiah, A. K., Wang, G. G. & Ma, Z. EPuL: an enhanced positive-unlabeled learning algorithm for the prediction of pupylation sites. *Molecules* **22**, 1463 (2017).

42 Sakai, T., Niu, G. & Sugiyama, M. Information-theoretic representation learning for positive-unlabeled classification. *Neural Comput.* **33**, 244–268. (2021).

43 Fernandez, A., Garcıa, S., Herrera, F. & Chawla, N. V. SMOTE for learning from imbalanced data: progress and challenges, marking the 15-year anniversary. *J. Artif. Intell. Res.* **61**, 863-905 (2018).

44 Chawla, N. V., Bowyer, K. W., Hall, L. O. & Kegelmeyer, W. P. SMOTE: synthetic minority over-sampling technique. *J. Artif. Intell. Res.* **16**, 321–357 (2002).

45 Iman, M., Rasheed, K. & Arabnia, H. R. A review of deep transfer learning and recent advancements. *Technologies* **11**, 40 (2023).

46 Elnaggar, A., Heinzinger, M., Dallago, C., Rehawi, G., Wang, Y., Jones, L., Gibbs, T., Feher, T., Angerer, C., Steinegger, M., Bhowmik, D. & Rost, B. ProtTrans: toward understanding the language of life through self-supervised learning. *IEEE Trans. Pattern Anal. Mach. Intell.* **44**, 7112-7127 (2022).

47 MacKay, D. J. C. *Information Theory, Inference, and Learning Algorithms*. (Cambridge University Press, 2003).

48 Breiman, L. Random Forests. *Machine Learning* **45**, 5–32 (2001).

49 Lundberg, S. M., Erion, G., Chen, H., DeGrave, A., Prutkin, J. M., Nair, B., Katz, R., Himmelfarb, J., Bansal, N. & Lee, S. I. From local explanations to global understanding with explainable AI for trees. *Nat. Mach. Intell.* **2**, 56-67 (2020).

50 Lundberg, S. M. & Lee, S. I. A unified approach to interpreting model predictions. *Adv. Neural Inf. Process. Syst.* **30**, 4768–4777 (2017).

51 Dickinson, Q. & Meyer, J. G. Positional SHAP (PoSHAP) for Interpretation of machine learning models trained from biological sequences. *PLoS Comput. Biol.* **18**, e1009736 (2022).

52 Gene Ontology Consortium, The Gene Ontology resource: enriching a GOld mine. *Nucleic Acids Res.* **49**, D325-D334 (2021).

53 Reimand, J., Isserlin, R., Voisin, V., Kucera, M., Tannus-Lopes, C., Rostamianfar, A., Wadi, L., Meyer, M., Wong, J., Xu, C., Merico, D. & Bader, G. D. Pathway enrichment analysis and visualization of omics data using g:Profiler, GSEA, Cytoscape and EnrichmentMap. *Nat. Protoc.* **14**, 482-517 (2019).

54 Raudvere, U., Kolberg, L., Kuzmin, I., Arak, T., Adler, P., Peterson, H. & Vilo, J. g:Profiler: a web server for functional enrichment analysis and conversions of gene lists (2019 update). *Nucleic Acids Res.* **47**, W191-W198 (2019).

55 Supek, F., Bosnjak, M., Skunca, N. & Smuc, T. REVIGO summarizes and visualizes long lists of gene ontology terms. *PLoS One* **6**, e21800 (2011).

56 Shannon, P., Markiel, A., Ozier, O., Baliga, N. S., Wang, J. T., Ramage, D., Amin, N., Schwikowski, B. & Ideker, T. Cytoscape: a software environment for integrated models of biomolecular interaction networks. *Genome Res.* **13**, 2498-2504 (2003).

57 Merico, D., Isserlin, R., Stueker, O., Emili, A. & Bader, G. D. Enrichment map: a network-based method for gene-set enrichment visualization and interpretation. *PLoS One* **5**, e13984 (2010).

58 Levi, H., Rahmanian, N., Elkon, R. & Shamir, R. The DOMINO web-server for active module identification analysis. *Bioinformatics* **38**, 2364-2366 (2022).
59 Szklarczyk, D., Gable, A. L., Lyon, D., Junge, A., Wyder, S., Huerta-Cepas, J., Simonovic, M., Doncheva, N. T., Morris, J. H., Bork, P., Jensen, L. J. & Mering, C. V. STRING v11: protein-protein association networks with increased coverage, supporting functional discovery in genome-wide experimental datasets. *Nucleic Acids Res.* **47**, D607-D613 (2019).
60 Doncheva, N. T., Morris, J. H., Gorodkin, J. & Jensen, L. J. Cytoscape StringApp: network analysis and visualization of proteomics data. *J. Proteome Res.* **18**, 623-632 (2019).
61 Assenov, Y., Ramirez, F., Schelhorn, S. E., Lengauer, T. & Albrecht, M. Computing topological parameters of biological networks. *Bioinformatics* **24**, 282-284 (2008).
62 Pinero, J., Ramirez-Anguita, J. M., Sauch-Pitarch, J., Ronzano, F., Centeno, E., Sanz, F. & Furlong, L. I. The DisGeNET knowledge platform for disease genomics: 2019 update. *Nucleic Acids Res.* **48**, D845-D855 (2020).
63 Johnson, M., Zaretskaya, I., Raytselis, Y., Merezhuk, Y., McGinnis, S. & Madden, T. L. NCBI BLAST: a better web interface. *Nucleic Acids Res.* **36**, W5-9 (2008).
64 Tikhomirov, O. & Carpenter, G. Caspase-dependent cleavage of ErbB-2 by geldanamycin and staurosporin. *J. Biol. Chem.* **276**, 33675-33680 (2001).
