# Supplementary material for: Charting γ-secretase substrates by explainable AI
Source: Nat Commun. 2025 Jul 1;16:5428. doi: 10.1038/s41467-025-60638-z (PMC12219630; doi:10.1038/s41467-025-60638-z)
Supplement: Supplementary file 2 — Description of Additional Supplementary Files [file 41467_2025_60638_MOESM2_ESM.pdf]

Legends Supplementary Data 1-23

Supplementary Data 1
Description: Summary of sequence datasets

Supplementary Data 2
Description: Sequence datasets (substrates, non-substrates, single-spanning proteins with unknown substrate status)

Supplementary Data 3
Description: α-Secretase substrates (based on literature reviews)

Supplementary Data 4
Description: β-Secretase substrates (based on literature, mainly mass spectrometry-based screens)

Supplementary Data 5
Description: All default CPP part-split combinations for APP and TMX3

Supplementary Data 6
Description: Scale subcategory selection (scale classification from AAontology)

Supplementary Data 7
Description: Scale set selections (Set 1-5) obtained using AAclust with agglomerative clustering (complete linkage)

Supplementary Data 8
Description: CPP features for 6 best dataset/annotation combinations, comparing SUBEXPERT vs OTHERS

Supplementary Data 9
Description: CPP features for different settings of *max_overlap* and *max_cor*

Supplementary Data 10
Description: Machine learning models and hyperparameters

Supplementary Data 11
Description: NONSUBPRED datasets

Supplementary Data 12
Description: Benchmarking results for CPP and dPULearn compared against ProtT5 embeddings (state-of-the-art protein language model)

Supplementary Data 13
Description: Substrate prediction scores for all single-span transmembrane proteins with JMD length ≥ 10

Supplementary Data 14
Description: Candidates for experimental validation

Supplementary Data 15
Description: CPP-SHAP analysis for APP, Notch2, and ITGB1 (for models trained on dataset 1 with TMHMM annotation)

Supplementary Data 16
Description: CPP-SHAP analysis clustering results (for models trained on dataset 1 with TMHMM annotation)

Supplementary Data 17
Description: Functional analysis: datasets with results

Supplementary Data 18
Description: Functional analysis: results of enrichment analysis

Supplementary Data 19
Description: Functional analysis: clustering of GO and pathway terms

Supplementary Data 20
Description: Functional analysis: new pathway/disease links (new HC substrates)

Supplementary Data 21
Description: Functional analysis: RNA expression (at tissue and single-cell level)

Supplementary Data 22
Description: Statistical analysis

Supplementary Data 23
Description: Overview

Remark: Tables **highlighted in bold** contain the key data/results of this study and are covered in the main text.
