# Supplementary material for: Charting γ-secretase substrates by explainable AI
Source: Nat Commun. 2025 Jul 1;16:5428. doi: 10.1038/s41467-025-60638-z (PMC12219630; doi:10.1038/s41467-025-60638-z)
Supplement: Supplementary file 4 — Reporting Summary [file 41467_2025_60638_MOESM4_ESM.pdf]

nature portfolio

Corresponding author(s): Harald Steiner
Dmitrij Frishman

Last updated by author(s): 02.05.2025

Reporting Summary

Nature Portfolio wishes to improve the reproducibility of the work that we publish. This form provides structure for consistency and transparency in reporting. For further information on Nature Portfolio policies, see our Editorial Policies and the Editorial Policy Checklist.

Statistics

For all statistical analyses, confirm that the following items are present in the figure legend, table legend, main text, or Methods section.

n/a	Confirmed	
☐	☒	The exact sample size (*n*) for each experimental group/condition, given as a discrete number and unit of measurement
☐	☒	A statement on whether measurements were taken from distinct samples or whether the same sample was measured repeatedly
☐	☒	The statistical test(s) used AND whether they are one- or two-sided<br>*Only common tests should be described solely by name; describe more complex techniques in the Methods section.*
☒	☐	A description of all covariates tested
☐	☒	A description of any assumptions or corrections, such as tests of normality and adjustment for multiple comparisons
☐	☒	A full description of the statistical parameters including central tendency (e.g. means) or other basic estimates (e.g. regression coefficient) AND variation (e.g. standard deviation) or associated estimates of uncertainty (e.g. confidence intervals)
☐	☒	For null hypothesis testing, the test statistic (e.g. *F*, *t*, *r*) with confidence intervals, effect sizes, degrees of freedom and *P* value noted<br>*Give P values as exact values whenever suitable.*
☒	☐	For Bayesian analysis, information on the choice of priors and Markov chain Monte Carlo settings
☒	☐	For hierarchical and complex designs, identification of the appropriate level for tests and full reporting of outcomes
☐	☒	Estimates of effect sizes (e.g. Cohen's *d*, Pearson's *r*), indicating how they were calculated

Our web collection on statistics for biologists contains articles on many of the points above.

Software and code

Policy information about availability of computer code

Data collection	All software used for data collection is described in detail in the Supplementary Information.<br>Protein sequence data were downloaded from the UniProtKB/Swiss-Prot database (https://www.uniprot.org/). Transmembrane domain annotations were obtained from UniProt and predicted by the TMHMM2 (https://services.healthtech.dtu.dk/services/TMHMM-2.0/) and Phobius (https://phobius.sbc.su.se/) web servers. Sequence redundancy was reduced using the CD HIT (https://sites.google.com/view/cd-hit) web server.<br><br>For the functional bioinformatics analysis, we retrieved the full STRING network (https://string-db.org/) using the Cytoscape (v3.9.1, https://cytoscape.org/) StringApp (v1.7.1, https://apps.cytoscape.org/apps/stringapp). We retrieved disease terms from the DisGeNET database (https://www.disgenet.org/) and natural variant mutation links from the UniProt database. The expression data for the human N-out proteome was obtained from the Human Protein Atlas database (v21.1, https://www.proteinatlas.org/) at the tissue and single cell levels.
Data analysis	All software used for data analysis, including detailed parameter settings and methodologies, is described in the Supplementary Information.<br>For the functional bioinformatics analysis, the initial GO term enrichment was performed using the g:Profiler web server (https://biit.cs.ut.ee/gprofiler/gost). GO and pathway terms were clustered using the REVIGO (http://revigo.irb.hr/) web server. Network analysis and visualization was performed using Cytoscape (v3.9.1, https://cytoscape.org/) with the following plugins: NetworkAnalyzer (v4.4.8, https://apps.cytoscape.org/apps/networkanalyzer), EnrichmentMap (v3.3.4, https://apps.cytoscape.org/apps/enrichmentmap), and AutoAnnotate (v1.3.5, https://apps.cytoscape.org/apps/autoannotate). Active protein modules were identified using the DOMINO web server (http://domino.cs.tau.ac.il/).

The novel algorithms (CPP, dPULearn) and visualizations (e.g., CPP profile, CPP feature map) are all available from our AAanalysis package, a Python-based framework for interpretable sequence-based protein prediction, documented in https://aaanalysis.readthedocs.io/en/latest/ and freely accessible at GitHub: https://github.com/breimanntools/aaanalysis. AAanalysis v1.0.0 was used for this study.

The computational analysis was performed with Python v3.9, using the following packages categorized by application:
- Data analysis: pandas v1.4.2, Numpy v1.22.4
- Statistical testing: SciPy v1.8.1, statsmodels v0.13.2
- Data visualization: matplotlib v3.5.2, seaborn v0.11.2
- Machine learning: scikit-learn v1.1.1, CatBoost v1.0.6, XGBoost v1.2.0
- Explainable AI: SHAP v0.40.0
- BLAST: Biopython v1.79

For manuscripts utilizing custom algorithms or software that are central to the research but not yet described in published literature, software must be made available to editors and reviewers. We strongly encourage code deposition in a community repository (e.g. GitHub). See the Nature Portfolio guidelines for submitting code & software for further information.

Data

Policy information about availability of data

All manuscripts must include a data availability statement. This statement should provide the following information, where applicable:

- Accession codes, unique identifiers, or web links for publicly available datasets
- A description of any restrictions on data availability
- For clinical datasets or third party data, please ensure that the statement adheres to our policy

All data generated in this study are openly and unrestrictedly available in the Supplementary Data. These include sequence datasets and related information (Supplementary Data 2–4, 11), CPP features and scale selections (Supplementary Data 5–9), a list of machine learning models and hyperparameters (Supplementary Data 10), substrate prediction scores for all single-spanning transmembrane proteins (Supplementary Data 13), an overview of experimentally validated substrates and non-substrates (Supplementary Data 14), results from the explainable AI analysis (Supplementary Data 15–16), findings from the functional bioinformatics analysis (Supplementary Data 17–21), and statistical testing results with exact p-values (Supplementary Data 22). An overview of these data is provided in Supplementary Data 23.

Our newly developed AAanalysis framework (https://github.com/breimanntools/aaanalysis) makes all amino acid scales and their two-level classification (AAontology) freely accessible. It also provides the TMHMM-based training data as a representative benchmarking dataset for protein prediction (https://aaanalysis.readthedocs.io/en/latest/index/tables.html).

Research involving human participants, their data, or biological material

Policy information about studies with human participants or human data. See also policy information about sex, gender (identity/presentation), and sexual orientation and race, ethnicity and racism.

Reporting on sex and gender	We did not perform sex- and gender-based analyses in this study, as they were not relevant to our research objectives and study design.
Reporting on race, ethnicity, or other socially relevant groupings	We did not perform analyses based on race, ethnicity, or other socially relevant groupings, as they were not relevant to our research objectives and study design.
Population characteristics	We did not analyze population characteristics, as they were not relevant to our research objectives and study design.
Recruitment	We did not conduct specific recruitment procedures, as they were not relevant to our research objectives and study design.
Ethics oversight	No ethical oversight was required for this study, as it did not involve human participants, animal subjects, or other ethical considerations.

Note that full information on the approval of the study protocol must also be provided in the manuscript.

Field-specific reporting

Please select the one below that is the best fit for your research. If you are not sure, read the appropriate sections before making your selection.

[x] Life sciences [ ] Behavioural & social sciences [ ] Ecological, evolutionary & environmental sciences

For a reference copy of the document with all sections, see nature.com/documents/nr-reporting-summary-flat.pdf

Life sciences study design

All studies must disclose on these points even when the disclosure is negative.

Sample size	The training dataset sizes were constrained by the availability of known γ-secretase substrates and non-substrates. The size for the substrate dataset was further refined based on sequence redundancy and the transmembrane domain annotations from UniProt, TMHMM, and Phobius.
Data exclusions	Single-span transmembrane proteins were excluded from the computational analysis if their N- and/or C-terminal juxtamembrane domain contained fewer than the number of residues required for CPP (set to 10). Amino acid scales lacking values for proline were omitted.
Replication	The number of machine learning training iterations was optimized to ensure reproducibility of prediction results. Cleavage assays for the experimental validations were performed at least three times based on good scientific practice for obtaining reproducible results.
Randomization	The training, test, and validation data were randomized during learning, and model evaluation followed a nested cross-validation approach. The control group of non-substrates used for the statistical evaluation of dPULearn was obtained through random sampling.
Blinding	As we were not aware of any potential sources of bias in our validation experiments, we were not blinded to allocation during candidate selection, experiment conduction, and outcome assessment.

Reporting for specific materials, systems and methods

We require information from authors about some types of materials, experimental systems and methods used in many studies. Here, indicate whether each material, system or method listed is relevant to your study. If you are not sure if a list item applies to your research, read the appropriate section before selecting a response.

Materials & experimental systems

n/a	Involved in the study	
☐	☒	Antibodies
☐	☒	Eukaryotic cell lines
☒	☐	Palaeontology and archaeology
☒	☐	Animals and other organisms
☒	☐	Clinical data
☒	☐	Dual use research of concern
☒	☐	Plants

Methods

n/a	Involved in the study	
☒	☐	ChIP-seq
☒	☐	Flow cytometry
☒	☐	MRI-based neuroimaging

Antibodies

Antibodies used	Rabbit monoclonal anti-His tag antibody RM146 (biotin conjugate) was obtained from NSJ Bioreagents (Catalog No. R20255BTN-50UG) and used at a dilution of 1:5,000. Mouse monoclonal anti-β-Actin antibody was obtained from Sigma (Product No. A5316, Batch number 123M4876) and used at a dilution of 1:10,000. Immuno Pure Goat anti-Biotin antibody was obtained from Pierce Biotechnology (Product number No. 31852, Lot number EG769216) and used at a final concentration of 1 µg/ml.
Validation	All commercial antibodies were validated by the manufacturer. As shown in Figure 5a,b and Supplementary Figure 7b,c, anti-His tag antibody specificity was further validated by immunoblotting, which confirmed the presence of immunoreactive bands for transfected His-tagged cDNA constructs and their absence in untransfected controls.

Eukaryotic cell lines

Policy information about cell lines and Sex and Gender in Research

Cell line source(s)	HEK293 cells stably expressing APP with the Swedish mutation (HEK293/sw) and a double knockout of presenilin 1 and presenilin 2 were described (Tagami et al., Cell Reports, 21 (2017) 259-273. doi:10.1016/j.celrep.2017.09.032).
Authentication	The absence of presenilin expression in these cells was confirmed by immunoblotting.
Mycoplasma contamination	Cells were not tested for mycoplasma contamination.
Commonly misidentified lines (See ICLAC register)	No commonly misidentified cell lines were used in the study.

Plants

Seed stocks	*Report on the source of all seed stocks or other plant material used. If applicable, state the seed stock centre and catalogue number. If plant specimens were collected from the field, describe the collection location, date and sampling procedures.*
Novel plant genotypes	*Describe the methods by which all novel plant genotypes were produced. This includes those generated by transgenic approaches, gene editing, chemical/radiation-based mutagenesis and hybridization. For transgenic lines, describe the transformation method, the number of independent lines analyzed and the generation upon which experiments were performed. For gene-edited lines, describe the editor used, the endogenous sequence targeted for editing, the targeting guide RNA sequence (if applicable) and how the editor was applied.*
Authentication	*Describe any authentication procedures for each seed stock used or novel genotype generated. Describe any experiments used to assess the effect of a mutation and, where applicable, how potential secondary effects (e.g. second site T-DNA insertions, mosiacism, off-target gene editing) were examined.*
